# Intron Retention in mRNA Encoding Ancillary Subunit of Insect Voltage-Gated Sodium Channel Modulates Channel Expression, Gating Regulation and Drug Sensitivity

**DOI:** 10.1371/journal.pone.0067290

**Published:** 2013-08-15

**Authors:** Céline M. Bourdin, Bénédicte Moignot, Lingxin Wang, Laurence Murillo, Marjorie Juchaux, Sophie Quinchard, Bruno Lapied, Nathalie C. Guérineau, Ke Dong, Christian Legros

**Affiliations:** 1 Laboratoire Récepteurs et Canaux Ioniques Membranaires (RCIM) UPRES EA 2647/USC INRA 1330, SFR QUASAV 4207, UFR Sciences, Université d'Angers, Angers, France; 2 Department of Entomology, Genetics and Neuroscience Programs, Michigan State University, East Lansing, Michigan, United States of America; 3 Laboratoire LIttoral ENvironnement et Sociétés (LIENSs), UMR 7266 CNRS, Institut du Littoral et de l'Environnement, Université de La Rochelle, La Rochelle, France; 4 IMAC, SFR QUASAV 4207, Université d'Angers, Beaucouzé, France; 5 Laboratoire de Biologie Neurovasculaire et Mitochondriale Intégrée, UMR CNRS 6214, INSERM 1083, UFR de Sciences Médicales, Université d'Angers, Angers, France; Xuzhou Medical College, China

## Abstract

Insect voltage-gated sodium (Na_v_) channels are formed by a well-known pore-forming α-subunit encoded by *para*-like gene and ancillary subunits related to TipE from the mutation “temperature-induced-paralysis locus E.” The role of these ancillary subunits in the modulation of biophysical and pharmacological properties of Na^+^ currents are not enough documented. The unique neuronal ancillary subunit TipE-homologous protein 1 of *Drosophila melanogaster* (DmTEH1) strongly enhances the expression of insect Na_v_ channels when heterologously expressed in *Xenopus* oocytes. Here we report the cloning and functional expression of two neuronal *DmTEH1*-homologs of the cockroach, *Periplaneta americana*, *PaTEH1A* and *PaTEH1B*, encoded by a single bicistronic gene. In PaTEH1B, the second exon encoding the last 11-amino-acid residues of PaTEH1A is shifted to 3′UTR by the retention of a 96-bp intron-containing coding-message, thus generating a new C-terminal end. We investigated the gating and pharmacological properties of the *Drosophila* Na_v_ channel variant (DmNa_v_1-1) co-expressed with DmTEH1, PaTEH1A, PaTEH1B or a truncated mutant PaTEH1Δ(270-280) in *Xenopus* oocytes. PaTEH1B caused a 2.2-fold current density decrease, concomitant with an equivalent α-subunit incorporation decrease in the plasma membrane, compared to PaTEH1A and PaTEH1Δ(270-280). PaTEH1B positively shifted the voltage-dependences of activation and slow inactivation of DmNa_v_1-1 channels to more positive potentials compared to PaTEH1A, suggesting that the C-terminal end of both proteins may influence the function of the voltage-sensor and the pore of Na_v_ channel. Interestingly, our findings showed that the sensitivity of DmNa_v_1-1 channels to lidocaine and to the pyrazoline-type insecticide metabolite DCJW depends on associated TEH1-like subunits. In conclusion, our work demonstrates for the first time that density, gating and pharmacological properties of Na_v_ channels expressed in *Xenopus* oocytes can be modulated by an intron retention process in the transcription of the neuronal TEH1-like ancillary subunits of *P. americana*.

## Introduction

Voltage-gated sodium (Na_v_) channels are critical for the initiation of action potentials (APs) in excitable cells [1]. Because of their crucial role in the regulation of membrane excitability in the nervous system, the Na_v_ channels are major targets of numerous natural toxins and therapeutic compounds, such as local anesthetics (LAs), antiepileptic drugs and antiarrythmics [2]. They also constitute a main molecular target of insecticides like pyrethroids and pyrazoline-type insecticides (PTIs) used in pest control [3], [4]. They consist in a pore-forming subunit (α-subunit) associated with ancillary subunits. In insects, two *para*-related genes encode α-subunits of Na_v_ channels [5], [6], sharing high sequence identity with those of vertebrate.

Insect auxiliary subunits of Na_v_ channels are structurally unrelated to well-known auxiliary β-subunits of vertebrate Na_v_ channels. While mammalian Na_v_ auxiliary β-subunits are single membrane-spanning proteins [7], *Drosophila melanogaster* auxiliary subunits (TipE and TEH1-4) contain two membrane-spanning segments [8], [9], [10]. In contrast with most mammalian Na_v_ α-subunit, expression of *para* (DmNa_v_1) alone generates very small Na^+^ currents in *Xenopus* oocytes [8],[9],[10]. The first insect auxiliary subunit described, TipE, strongly enhances Na^+^ currents when co-expressed with DmNa_v_1 in *Xenopus* oocytes, indicating that these proteins have strong chaperone or stabilizing effects on channel expression [8], [9], [10]. More recently, Derst et al. [8], show that TEH1 displays a stronger expression stimulating effect than TipE on DmNa_v_1 expression. By contrast, TEH2 and TEH3 stimulate channel expression to a lower extent, while TEH4 does not at all. Co-expression of DmNa_v_1 and TipE results in Na^+^ currents with same activation properties, but with accelerated current decay and also faster rate of recovery from inactivation [8], [10]. Moreover, Na^+^ currents elicited by co-expression of DmNa_v_1 channels and TipE or TEH1-4, display specific inactivation and repriming properties, reminding functional properties of mammalian β-subunits [8], [10].

In *D. melanogaster*, TEH1 was found exclusively expressed in neuronal tissues, while TipE, TEH2 to TEH4 have a broad tissue distribution, including non-excitable cells [8]. This suggests that TEH1 and TEH1-like proteins of other insect species are relevant elements in the modulation of neuronal excitability through the modulation of the gating and pharmacological properties of Na_v_ channels. Indeed, TEH1 dramatically changes the inactivation properties (voltage-dependence and recovery from inactivation) of Na^+^ currents generated by DmNa_v_1 channels in *Xenopus* oocytes in comparison to TipE [8]. Thus, we hypothesized that DmNa_v_1/TEH1 channels display different pharmacological properties compared to DmNa_v_1/TipE channels.

PTIs belong to a relatively new class of insecticides, including indoxacarb and metaflumizone which kill a broad spectrum of insects by inhibiting Na_v_ channels [11]. Indoxacarb acts as a proinsecticide, which is biotransformed into a toxic N-decarbomethoxyllated metabolite DCJW [12]. DCJW irreversibly blocks APs in preparations of lepidopteran (*Manduca sexta*) larval motor nerve and induces a dose-dependent inhibition of Na_v_ channels in cockroach *Periplaneta americana* neurons [13], [14]. The similarity between the blocking mechanism induced by PTIs and LAs, such as lidocaine was early reported [15]. Indoxacarb/DCJW and lidocaine preferably bind to inactivated channels resulting in stabilization of this state [13], [14], [15], [16], [17], [18]. Since PTIs potency depends on the inactivation properties of Na_v_ channels [13], [14], [15], TEH1 could also differentially modulate the sensitivity of Na_v_ channel to insecticides.

The cockroach model is a usual tool to investigate whether and how do neurotoxic insecticides modulate ion channels. *P. americana* is suitable neurobiological model, for which electrophysiological and pharmacological studies have shown the existence of different Na^+^ currents with distinct biophysical and pharmacological properties in isolated neurons [14], [19], [20]. To gain further insights into the molecular characterization of Na_v_ channels in *P. americana*, we cloned and characterized two novel variants of *teh1-like* gene. We showed that these TEH1-like variants are exclusively expressed in the nervous system of *P. americana* and resulted from exclusion or retention of an intron that modulates expression and gating properties of the DmNa_v_1-1 variant expressed in *Xenopus* oocytes. Furthermore, we examined the biophysical properties of Na^+^ currents resulting from the co-expression of DmNa_v_1-1 with TEH1 and TEH1-like variants of *P. americana*. Next, we characterized the pharmacological consequences of the co-expression of these auxiliary subunits and we observed marked differences in the blocking effects of lidocaine and DCJW on DmNa_v_1-1 channels.

## Materials and Methods

### Insects

 Adult male American cockroaches *P. americana* were obtained from our laboratory stock colony maintained at 29°C on 12-h light/dark cycle with food and water *ad libitum*. Intestine, ganglia, reproductive glands, muscles (extracted from the coxa of the legs), head and the ventral nerve cord (thoracic and abdominal ganglia, together with the connectives) were dissected from adult male cockroaches.

### RNA extraction, RT-PCR and cloning of full length PaTEH1 cDNAs

Total RNA extraction was isolated from various tissues using the TRIzol® Reagent (Ozyme/Biogentex, France). For polymerase chain reaction (PCR) experiments, first strand cDNAs were synthesized from 5 µg of total RNA using SuperScriptTM III First-Strand Synthesis System Super Mix (Invitrogen, USA) in the presence of oligo (dT)_20_. The sequences of the primers used in our experiments are listed in Table 1. For cloning PaTEH1 cDNA in the nerve cord, a degenerated primers pair (DPS1 and DPR1) was designed from conserved regions observed in an alignment of available TEH1 subunit sequences. A PCR amplification of cDNA fragment was carried out using EuroTaq polymerase (Eurobio, France). PCR products were separated by agarose gel electrophoresis and purified using Nucleospin® Gel and PCR clean up (Macherey-Nagel, Germany). The cDNA fragments were cloned into PCR® 4 TOPO® (Invitrogen). Each clone was sequenced in both strands by the company Millegen Biotechnology. For RACE (rapid amplification of cDNA ends) of 5′ and 3′ ends of PaTEH1, the RNA ligase-mediated rapid amplification of cDNA ends method (GeneRacer™ kit, Invitrogen) was used. For amplification of 5′end and 3′end regions, the gene-specific primer used were P-R1, P-R2, P-S1 and P-S2. PCR products were cloned and sequenced as described above. Finally, the ORF of PaTEH1 cDNAs was amplified using the gene-specific primers P-S3 and P-R3, containing the restriction enzyme sites *Xma*I and *Xba*I respectively, for directional cloning into pGEM-HEJUEL plasmid suitable for expression in *Xenopus* oocytes (kindly provided by Pr. Olaf Pongs, Institute for Neural Signal Transduction, Hamburg, Germany). Sequences analyses were performed using BioEdit sequence analysis Software. Full- length ORFs were identified using BLAST research in the GenBank database (http://www.ncbi.nlm.nih.gov/blast/Blast.cgi). Amino acid sequences alignment was carried out using ClustalW method as described [6].

**Table 1:** Sequences of the oligonucleotides used in PCR and their corresponding region.

Primers name	Nucleotide sequence	Domain
1-Degenerated primer used to amplify PaTEH1		
DPS1	5′-GTGCCGCTSTACGTSGAYCCS-3′	MS1–MS2
DPR1	5′-ATGCATTGYGACTGCMGSTG-3′	MS1–MS2
2-Specific primers used in RACE		
P-R1	5′-CGATGGGGATGTTGATGTTGAC-3′	5′UTR partial ORF PaTEH1(PCR#1)
P-R2	5′-CGTACATGGTGGTGAAGTTGTC-3′	5′UTR partial ORF PaTEH1(PCR#2)
P-S1	5′-GTCAACATCAACATCCCCATCG-3′	3′UTR partial ORF PaTEH1(PCR#1)
P-S2	5′-GACAACTTCACCACCATGTACG-3′	3′UTR partial ORF PaTEH1(PCR#2)
3-Primers used to amplify the full-length ORF		
P-S3	5′-CAGATCCCGGGATGAGGAGCAGCAGCTCGGAG-3′	Full-length ORF PaTEH1
P-S4	5′-AAAAGTTGTCAGCTGTTCGGCG-3′	Complete cDNA PaTEH1
P-R3	5′-CTGATTCTAGACCGAATCATGTTCTATCTTCT-3′	Full-length ORF PaTEH1
P-R4	5′-(T)_23_ CAAAATATAGGCCATGTATTTCTACC-′3	Complete cDNA PaTEH1

Designation of oligonucleotide mixtures: S = G+C; Y = C+T; M = A+C.

UTR, untranslated region; ORF, open-reading frame.

Restriction enzyme recognition sequences are underlined and correspond to *Xma*I site (CCCGGG) and *Xba*I site (TCTAGA).

Semi-quantitative RT-PCR experiments were performed to study the tissue distribution pattern of PaTEH1 using the P-S3/R3 primer pair. The experiments were repeated five times.

### Extraction of genomic DNA and PCR

Genomic DNA was extracted from five cockroach individuals using the procedure modified from Blin and Stafford [21]. Tissues were frozen in liquid nitrogen and stored at −80°C. Frozen tissues were homogenized in an extraction buffer (1 ml per 100 mg tissue) containing Tris-Cl 10 mM (pH 8.0), EDTA 0.1 M (pH 8.0), pancreatic RNAase 20 µg/ml, 0.5% SDS and incubated for 1 h at 37°C. Proteinase K was added to a final concentration of 100 µg/ml and the suspension was incubated at 50°C for 3 h. Three phenol extractions were carried out and the DNA in aqueous phase was precipitated with 0.2 volume of 10 M acetate ammonium and 2 volumes of ethanol. High molecular weight genomic DNA was recovered with a Pasteur pipette. DNA was dissolved in TE (pH 8.0) and stored at 4°C. *Pateh1* gene was amplified by nested PCR using the P-S4/R4 primer pair, followed by the P-S3/R3 primer pair with genomic DNA as template. The amplified product of the second PCR was cloned and sequenced as described above.

### Co-expression of DmTEH1 or PaTEH1 variants with DmNa_v_1.1 in Xenopus laevis oocytes

A splice variant of the sodium channel from *D. melanogaster*, DmNa_v_1.1, was used for co-expression experiments [22]. pGEM-DmTEH1 construct was kindly provided by Dr Christian Derst (Institute for Integrative Neuroanatomy, Charité, Berlin, Germany). All recombinant plasmids were linearized with *Spe*I (Promega, Madison, USA) or *Not*I (Invitrogen), and capped RNA were transcribed in vitro using the T7 mMESSAGE mMACHINE kit (Ambion, Austin, USA).

Ovarian lobes were surgically harvested from adult *Xenopus laevis* previously anesthetized in tricaine (0.15 M, Sigma-Aldrich) and washed in a standard oocyte saline (SOS) solution composed of (in mM): NaCl 100, KCl 2, CaCl_2_ 1.8, Hepes 5, pH 7.5. Defolliculated oocytes were obtained after 1 h-treatment with 2 mg/ml collagenase (type 1A, Sigma-Aldrich, Saint Quentin Fallavier, France) and 0.8 mg/ml trypsin inhibitor (Sigma-Aldrich) in calcium-free SOS. Depending on the combination of DmNa_v_1-1 and auxiliary subunits, various amounts (2 to 35 ng) of DmNa_v_1-1 and auxiliary subunits RNA mixture were injected in oocytes (1∶1 ratio). Injected oocytes were incubated in sterile medium composed of SOS supplemented with gentamycin (50 µg/ml), penicillin (100 U/ml), streptomycin (100 µg/ml) and sodium pyruvate (2.5 mM) at 18°C for 2 to 10 days before recordings.

### Ethics Statement

This study was carried out in strict accordance with the recommendations in the Guide for the Care and Use of Laboratory Animals of the European Community. The protocol was approved by the «Direction Départementale des services Vétérinaire du Maine et Loire» (N°B49071) and by the “Comité d'éthique en expérimentation des Pays de la Loire» (N°CEEA.2012.68).

### Electrophysiological recordings and analysis

Na^+^ currents were recorded using the two electrode voltage-clamp (TEVC) technique. Oocytes were tested using 1 M-KCl/2 M-Kacetate-filled borosilicate glass electrodes connected to a TEVC amplifier (TEV-200, Dagan Corporation, Minneapolis, Minessota, USA). Digidata 1440A interface (Axon CNS Molecular Devices, California, USA) and pCLAMP 10 software (Axon CNS Molecular Devices) were used for acquisition (sampling rate: 10 or 100 kHz; band pass-filter: 5 kHz) and stimulation protocols. All experiments are performed at room temperature (18–21°C) in SOS. Capacitive transient and leak currents were corrected using P/N (N = 6) subtractions. For activation experiments, families of Na^+^ current traces were generated by step depolarisations to test potentials from −70 to +40 mV (10 mV increment) from a holding potential of −100 mV. Na^+^ channel conductance (G) was calculated using the following equation G = I/(V−V_rev_) where I is the current amplitude, V is the test potential and V_rev_ is the reverse potential [23]. The reverse potential was determined from I/V curve fitted using Stühmer's equation: I = G*(1−(1/(1+exp((V−V_1/2_)/K))))*(V−V_rev_) [23]. Conductance values (G) were normalized to the maximum conductance and plotted against the test voltage. The resulting curves were fitted with the following Boltzmann's equation G = 1/(1+exp((V_1/2_−V)/k)) where V_1/2_ is the potential at which half of the Na_v_ channels are activated and k is the slope factor [19]. To determine the voltage dependence of steady state fast inactivation, families of Na^+^ current traces were generated by a two-pulse protocol beginning with a step of potential ranging from −80 mV to +40 mV (5 mV increment) for 200 ms. A second 12 ms test pulse was applied to −5 mV, a potential at which the current amplitude was maximal. Each set of double pulses was separated by 10 s. For determination of the voltage dependence of steady-state slow inactivation, a two-pulse protocol was used, starting with a first step of potential ranging from −90 mV to +20 mV (10 mV increment) for 60 s, followed by a 100 ms interval at the holding potential. A second pulse to −5 mV for 10 ms allowed measuring the available current. For both fast and slow inactivation, the peak current recorded during the second pulse was normalized to the maximum current peak and the data were fitted with Boltzmann's equation I/Imax = 1/(1+(exp((V−V_1/2_)/k)) where V_1/2_ is the potential at which half of the Na_v_ channels are inactivated and *k* is the slope factor [23]. For recovery from inactivation experiments, Na^+^ currents were generated by a two-pulse protocol beginning with a first pulse to −5 mV for 50 ms followed by a second pulse to −5 mV for 14.5 ms. The duration between the two pulses was increased by 1 ms every set of double pulses and each set was separated by 10 s. Fractional recovery was calculated by dividing the maximum current amplitude the second test pulse by the maximum current amplitude of the corresponding first pulse. The recovery data were fit with the following exponential equation: I = I_0_+(Imax−I_0_)*(1−exp (−k*(t−t_0_))) where *k* is the rate constant and t is the time (GraphPad Software; equation name: plateau followed by one phase association equation).

### DCJW and lidocaine sensitivity assays

Oocytes were perfused with 2 µM DCJW (DPX-JT333, according to a Material Transfer Agreement between Angers University and DuPont Crop Protection, Newark, USA) for 45 min. Before DCJW was perfused, several stable recordings were performed. Oocytes were perfused with 2 mM lidocaine (Sigma-Aldrich) for 10 min and Na^+^ channel blockade was measured at −5 mV. Then the ratio of peak Na^+^ current after lidocaine perfusion was measured and compared to the control peak. Protocols described above for activation, slow and fast steady-state inactivation and recovery from fast inactivation were also performed after drug application.

### Protein expression analysis using confocal laser scanning microscopy

For confocal microscopy experiments, albinos *Xenopus* oocytes were used. To detect auxiliary subunits, a histidine tag (RGHHHHHH) was introduced by PCR at the 5′ end of PaTEH1A and PaTEH1B constructs. Five days after injection with water or with 3 ng RNA encoding DmNa_v_1.1 and PaTEH1A or PaTEH1B, oocytes were fixed in phosphate buffered saline (PBS) containing 2% paraformaldehyde for 30 min at 4°C. Fixed oocytes were washed in PBS and blocked in PBS-Triton (PBS-T) 0.2% fish gelatine for 1 h at RT. Then, oocytes were incubated 10 h at 4°C with mouse RGS-His antibody (dilution 1∶500; Qiagen) and rabbit Anti-Pan Na_v_ (SP19) antibody (1∶100; Euromedex, France). Oocytes were washed three times in PBS-T 0.2% for 5 min and were next incubated 90 min with Goat Anti-Mouse IgG Alexa Fluor® 546 antibody and Goat Anti-Rabbit IgG Alexa Fluor® 488 (dilution 1∶3000, Invitrogen, USA) and washed three times in PBS. Oocytes were placed in dishes (ibidi, Martinsried, Germany) in PBS/glycerol 5∶1 solution and imaged through the ×10/0.3 numerical aperture objective of a Nikon (Nikon Instruments, Melville, NY) A1S1 confocal laser scanning microscope equipped with argon-ion (488 nm) and diode (561 nm) lasers. Alexa Fluor® 488 was excited at 488 nm and the fluorescence emission was collected using a 500–550 nm filter; Alexa Fluor® 546 was excited at 561 nm and the fluorescence emission was collected using a 570–600 nm filter. ImajeJ software (version 1.4.3.67) was used to measure relative fluorescence. To quantify relative fluorescence intensity for each tested oocyte, an identical area of 5002.2 µm^2^ was delimited and the total number of pixels corresponding to AlexaFluor® 488 or AlexaFluor® 546 in this area was determined. No fluorescence was measured for controls in which primary antibodies were omitted. Control of immunostaining specificity was performed using the same procedure in separate experiments: first with rabbit Anti-Pan Na_v_ (SP19) and secondary Goat Anti-Mouse IgG Alexa Fluor® 546 antibodies, and second with mouse RGS-His and secondary Goat Anti-Rabbit IgG Alexa Fluor® 488 antibodies.

### Statistical analysis

Statistical analysis of all data was performed using GraphPad Prism software (version 5). All data are presented as means ± standard error of the mean (SEM). Significance tests between groups of data were performed using a variance analysis (one-way ANOVA) followed by a Tukey post hoc test for comparison of all groups, or the unpaired Student's *t*-test. *p* values are specified. Statistical probabilities are expressed as * p<0.05, ** p<0.01 and *** p<0.001.

## Results

### Analysis of cDNA clones unmasks possible intron retention in PaTEH1 transcript

**Figure 1:**
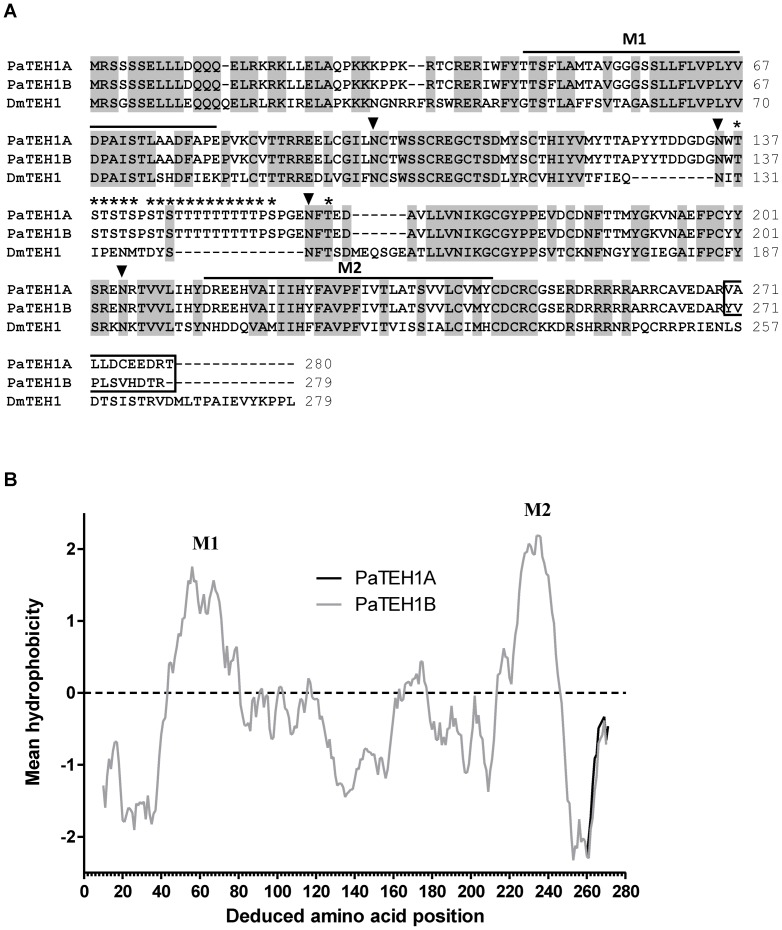
Primary structure analysis of TEH1-like auxiliary subunits of *P. americana*. A. Clustal W alignment of PaTEH1A (GenBank accession number KC206367), PaTEH1B (accession number KC206368) and DmTEH1 (accession number NP_649959). Transmembrane segments (M1 and M2) are indicated with bold line above the sequences. Conserved N-glycosylation sites are indicated by closed inverted triangles (▾) and O-glycosylation sites of PaTEH1s subunits are indicated by asterisk (*) (www.cbs.dtu.dk). Gaps are indicated by dashes. The undecapeptide (VALLDCEEDRT) and the decapeptide (YVPLSVHDTR) at the C-terminal ends of PaTEH1 variants are boxed. B. Hydrophobicity profile and deduced topological organization of PaTEH1A and PaTEH1B. Hydrophobicity analysis was performed using the algorithm of Kyte and Doolittle (1982). The amino acid residue position is plotted along the x-axis and the calculated mean hydrophobicity is plotted along the y-axis. Regions above the line are hydrophobic. The two putative membrane-spanning segments are indicated as M1 and M2.

Since the genome sequence of *Periplaneta americana* is not available in databases, a conventional RT-PCR strategy was performed to clone the cDNAs encoding the homolog of DmTEH1 in the nerve chord of *P. americana*. Briefly, using degenerated primers P-S1 and P-R1 (Table 1) designed from conserved amino acid regions deduced from an alignment of TEH1 subunits of *Drosophila melanogaster* (Genbank accession number NP_649959), *Anopheles gambiae* (accession number XP_310354), *Apis mellifera* (accession number XP_001120804), *Nasonia vitripennis* (accession number XP_001606835) and *Tribolium castaneum* (number XP_969821), a 561 bp cDNA fragment was amplified. Blast analysis indicated that the deduced amino acid sequence of this cDNA shares 51% of sequence identity with the extracellular loop of DmTEH1 and it was thus identified as TEH1-like protein of *P. americana* (PaTEH1). This partial *PaTEH1* cDNA allowed us to design specific primers for 5′ and 3′ RACE experiments. By 5′ RACE experiment, we obtained a cDNA fragment of 986 bp containing 432 bp of the ORF and 554 pb of the 5′ untranslated region (5′UTR). 3′RACE amplification led to the identification of two cDNA fragments of 618 bp and 712 bp. The sequence of the long fragment differed from that of the short one only by 94 bp insertion 37 bp upstream the stop codon of the short fragment. This indicated the presence of two transcripts with distinct ORF 3′ends. The 618 bp fragment contained the last 197 bp of the first ORF; followed by a 421 bp 3′UTR, and the 712 bp fragment contained the last 194 bp of the second ORF, followed by 518 bp 3′UTR. Both 3′UTR sequences exhibited polyA tail without the consensus ‘AATAAA’ polyadenylation site. Finally, using a primer pair encompassing the ORF, we amplified two different cDNAs. Ten clones contained an ORF of 843 bp encoding a 280 amino acids protein, named PaTEH1A (accession number KC206367). Six other clones displayed a longer insert (937 bp), containing an ORF of 840 bp encoding a 279 amino acids protein, named PaTEH1B (accession number KC206368). The nucleotide sequence of *PaTEH1B* clones corresponded to *PaTEH1A* but with an insertion of 94 bp after the position 807. This 94 bp insert in *PaTEH1B* sequence contained a novel stop codon and modified the coding sequence of the 3′end, leading to a deduced amino acid protein, PaTEH1B that diverged from PaTEH1A sequence only by the last 11 residues of their C-termini (Figure 1A). These data indicated that *PaTEH1A* and *PaTEH1B* likely result from an alternative splicing mechanism of a single gene.

Hydrophobicity analysis [24] of amino acid sequences of PaTEH1A and PaTEH1B (Figure 1B) revealed two hydrophobic domains (from positions 40 to 67 and 225 to 243) that aligned with the putative transmembrane domain of DmTEH1. Both hydrophobic profiles were overlapped up to the amino acid residue 269. The three PaTEH1 variants contained four putative N-glycosylation sites (Asn residues at positions 98, 135, 161 and 205) common with DmTEH1 (Asn residues at positions 101, 129, 142 and 191) (Figure 1A). Twenty putative O-glycosylation sites (Thr residues at positions 137, 139, 141, 145, 147 to 155 and 163 and Ser residues at positions 138, 140, 142, 144, 146 and 157) were detected in the region between the two membrane-spanning domains of PaTEH1 variants (Figure 1A).

**Figure 2:**
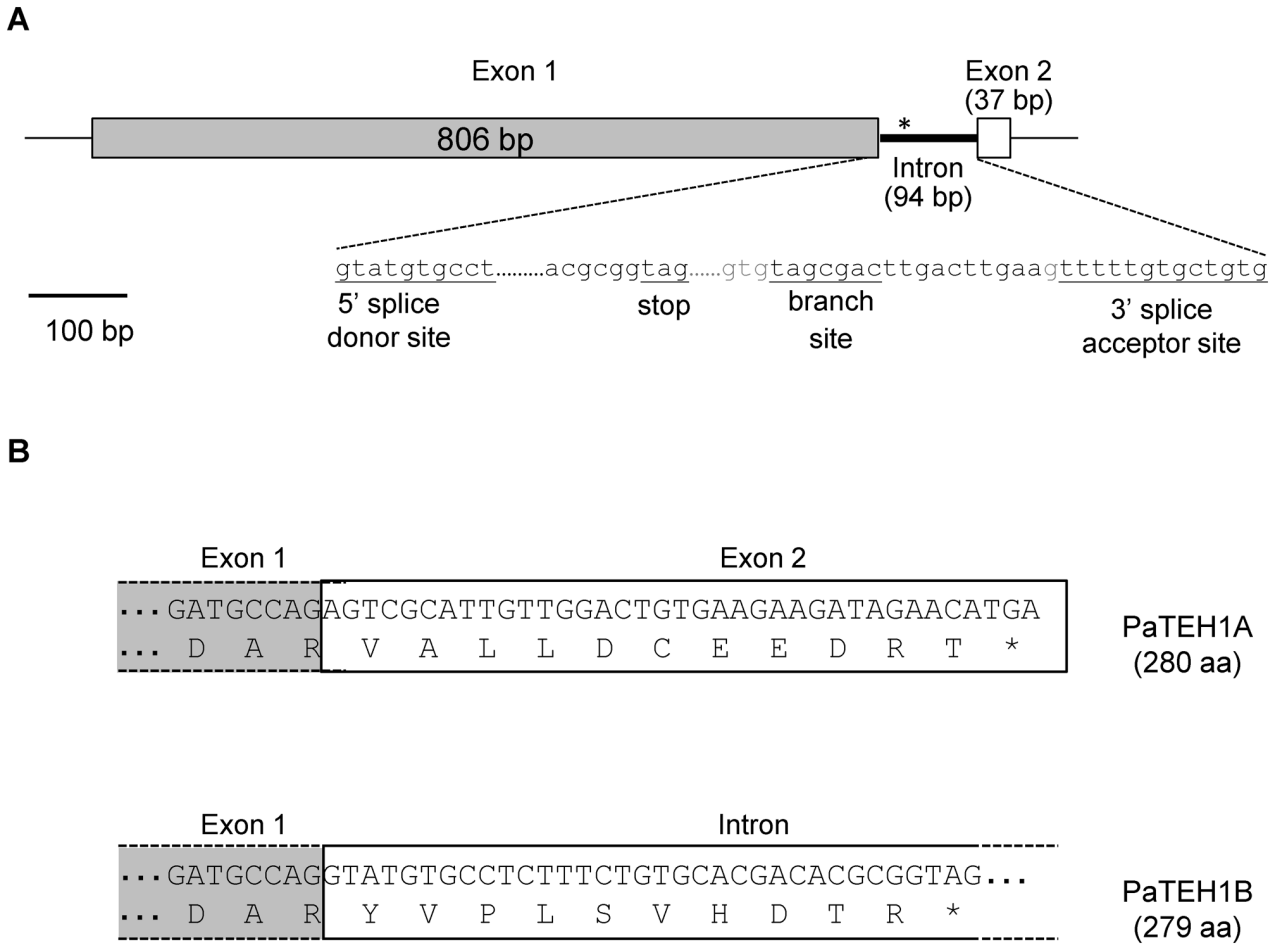
Organization of genomic region and mRNA and encoding TEH1-like subunit of *P. americana*. A. The genomic region of *Pateh1* consists in two exons interrupted by a short intron. The intron sequence is in lowercase letters and splice donor site, branch site and splice acceptor site are underlined. Sizes of exons and intron are indicated in parentheses. *: stop codon. B. Exclusion or retention of the intron led to PaTEH1A and PaTEH1B variants with different C-terminal ends. The 94 bp intron sequence contains a short new coding sequence followed by an in-frame stop codon. This generates a second protein (PaTEH1B) with a novel C-terminal end.

To determine whether *PaTEH1A* and *PaTEH1B* were the result of an alternative splicing event, a PCR was carried out using genomic DNA of *P. americana* and primers encompassing the ORF of *PaTEH1A*. A single 900 bp PCR fragment was cloned and its sequence totally matched with the sequence of *PaTEH1B* cDNA. A 94 bp insert, which was not present in *PaTEH1A*, found in this partial *Pateh1* gene sequence was delimited by donor and acceptor splice sites (Figure 2A). While the 5′ splice site sequence conformed to the consensus sequence that is known to promote splicing, the 3′ splice site sequence (tttttgtgctgtg) did not [25]. This 3′ splice site was considered as weak since it contained three unusual “G” (at positions −8, −6 and −3) and it ended with “TG” instead of the highly conserved dinucleotide “AG” (Figure 2A). The unusual TG splice acceptor was reported in both insect and mammalian genes [26], [27]. The additional 94 bp sequence found in *Pateh1* gene also showed a consensus branch site (TAGCGAC) close to the end of the putative intron. In conclusion, assuming that only one copy of *teh1* gene is present in *P. americana* genome, the 94 bp insert most likely corresponds to an intron that is unspliced in *PaTEH1B* (Figure 2B). Altogether, these observations demonstrate that the variant *PaTEH1B* results from an intron retention mechanism.

### Tissue distribution of PaTEH1 transcripts

**Figure 3:**
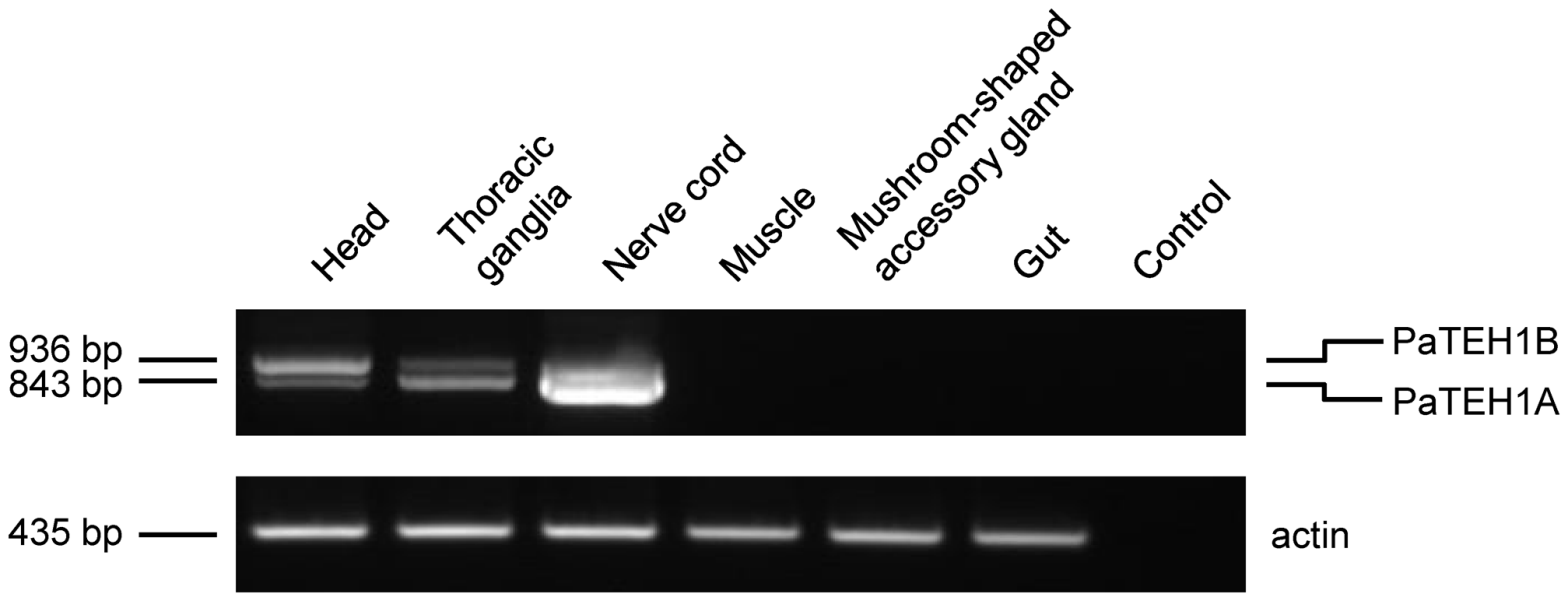
Semi-quantitative RT-PCR analysis of PaTEH1 expression in various *P. americana* tissues. RT-PCR was performed using 5 µg of mRNA extracted from head, thoracic ganglia, nerve cord, muscles, gut and mushroom-shaped accessory gland. Actin (Genbank accession number AY116670) was used as internal quantitative control.

Semi-quantitative RT-PCRs using P-S3 and P-R3 primers encompassing the ORF were performed to determine the expression pattern of *PaTEH1* transcripts, in six different tissues. First, amplification of *PaTEH1* cDNAs were detected in head, thoracic ganglia and nerve cord (Figure 3), indicating that *PaTEH1* variants were exclusively expressed in neuronal tissues. The agarose gel showed two bands with different intensity. The lower band matched perfectly with *PaTEH1A* (expected size: 843 bp) and the upper band exactly corresponded to *PaTEH1B* (expected size: 936 bp). *PaTEH1A* transcripts appeared to be less abundant in head than *PaTEH1B*. By contrast, *PaTEH1A* transcripts were expressed at higher levels than *PaTEH1B* both in thoracic ganglia and in nerve cord.

### Na^+^ current densities are modulated by PaTEH1 variants in *Xenopus* oocytes

**Figure 4:**
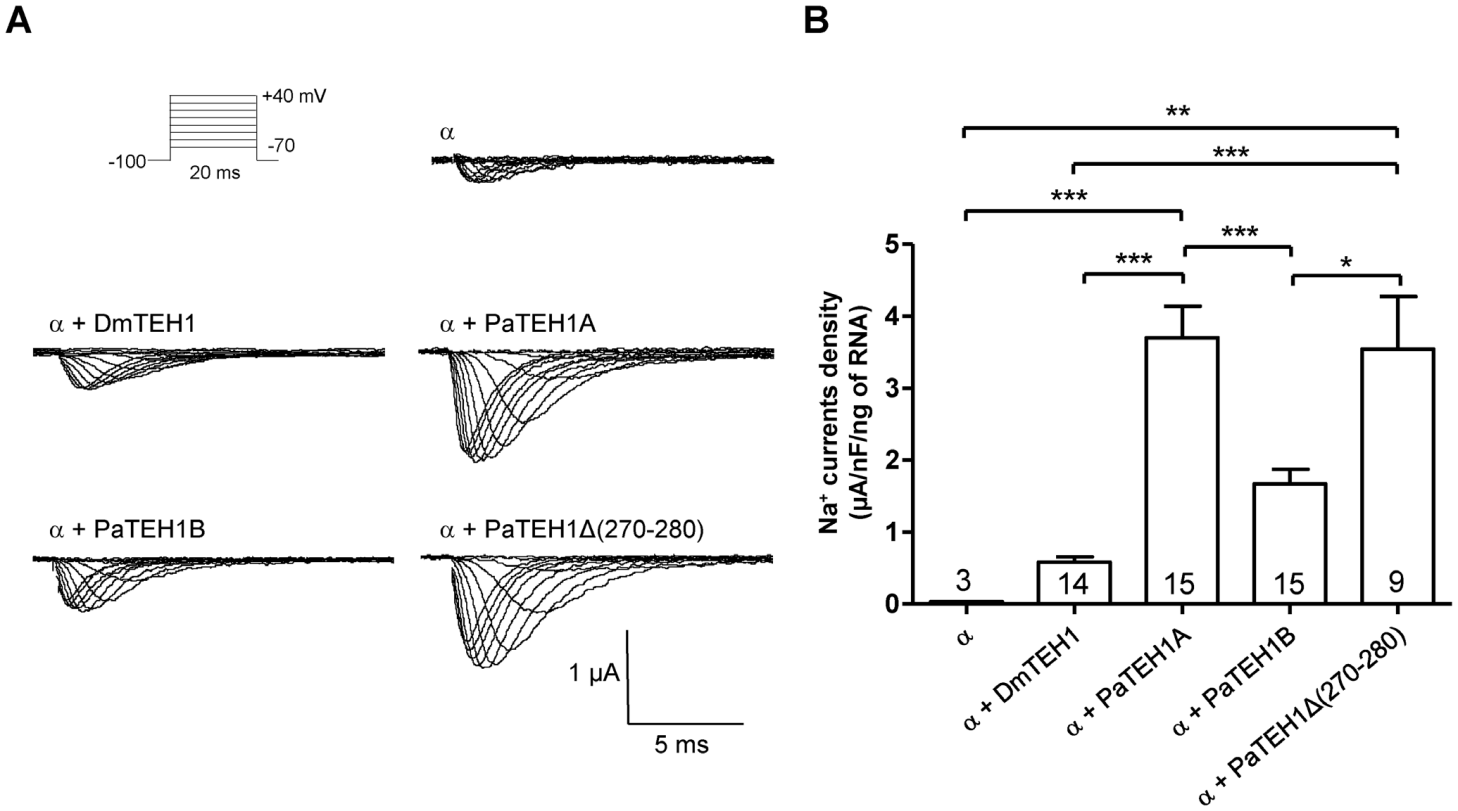
Modulation of Na^+^ current densities by different TEH1 auxiliary subunit subtypes. A. Family of Na^+^ currents measured at test potentials of −70 mV to 40 mV from a holding potential of −100 mV for DmNa_v_1-1 alone (α) or co-expressed with DmTEH1 (α+DmTEH1), PaTEH1A (α+PaTEH1A), PaTEH1B (α+PaTEH1B) or PaTEH1Δ(270-280) (α+PaTEH1Δ(270-280)) injected with about 35 ng, 8 ng, 2 ng, 3 ng and 2 ng of RNA (α∶β, 1∶1), respectively. The protocol of depolarizing voltage-clamp steps is shown. B. Na^+^ current density per ng of injected RNA after 3-days incubation, except for DmNa_v_1-1 (10 days after injection). Results are expressed in µA per nF per ng of injected RNA (One-way ANOVA: F_(4,55)_ = 15.11, p<0.0001 post hoc Tukey test). The number of tested oocytes is indicated in the bar histogram.

We first characterized the Na^+^ currents elicited by the expression of DmNa_v_1-1 [22], alone or in combination with DmTEH1, PaTEH1A and PaTEH1B in *Xenopus* oocytes (Figure 4). No current was measurable in oocytes injected with DmTEH1, PaTEH1A and PaTEH1B alone as control (data not shown). We observed that the required amounts of RNA (1.8 to 35 ng) to record Na^+^ currents with appropriate amplitudes (with maximum peak comprised between 0.25 and 2 µA) varied in each case. Unfortunately, we were unable to compare these effects with the co-expression of the Na_v_ α-subunit of *P. americana* [6] and DmTEH1, DmTipE, PaTEH1A or PaTEH1B, because Na^+^ currents were not detectable even after prolonged incubation time (Figure S1). As illustrated in Figure 4A, 35 ng of DmNa_v_1-1 RNA were injected in oocytes to obtain a maximum peak of ∼0.25 µA after ten days without any auxiliary subunits. On the other hand, when co-injected with DmTEH1, 4.2-fold fewer DmNa_v_1-1 RNAs were needed to yield 0.4 µA maximum current after 3-days incubation, indicating a chaperone-like effect that had been previously reported [8]. Interestingly, this effect was significantly stronger with PaTEH1A (1.2 µA, 3-days incubation) and PaTEH1B (0.6 µA, 4-days incubation), even for lower amounts of injected RNAs (1.8 and 2.8 ng, respectively). For an accurate comparison of the chaperone-like effect of these different β-subunits, the current densities of Na^+^ currents were measured as a ratio of maximum current density and RNA amount after 3 days of injection (Figure 4B). In comparison to DmTEH1, PaTEH1A had the strongest chaperone-like effect (6.3-fold, p<0.001), followed by PaTEH1B (2.8-fold, p<0.001). We also observed a decrease in the proportion of oocytes expressing detectable currents with PaTEH1B (∼50%) compared with PaTEH1A (∼90%). PaTEH1A appeared to be more efficient (2.2-fold, p<0.001) than PaTEH1B to increase Na^+^ current densities. Since both differed in sequence only by their last 11 amino acids residues, the unique modification by PaTEH1B was the result of the novel C-terminal extremity generated by the intron retention mechanism. A truncated form of PaTEH1s lacking the 11 last amino-acid residues (PaTEH1Δ(270-280)) modulated Na^+^ current densities similarly to PaTEH1A (Figure 4B). The high sequence identity shared by DmNa_v_1 with PaNa_v_1 (81%) and with the Na_v_ channel of the German cockroach *Blatella germanica*, BgNa_v_1 (77%) [6] led us to postulate that the consequence of the interaction between α-subunit and auxiliary subunit would be conserved in insects. To confirm this assumption, we co-expressed the variant BgNa_v_1-1a, containing a similar exon organization as DmNa_v_1-1 with PaTEH1A or PaTEH1B (Figures S2). The resulting Na^+^ current densities were roughly twice-fold higher in oocytes expressing BgNa_v_1-1a/PaTEH1A than BgNa_v_1-1a/PaTEH1B, arguing that the C-terminal end of PaTEH1B modulated *Drosophila* and cockroach Na_v_ channel expression to a similar extent. Altogether, our data indicate that the last 11 amino-acid residues of PaTEH1A are not involved in the chaperone-like effects and the presence of the unique C-terminal sequence in PaTEH1B encoded by the retained intron interferes with the chaperone-like properties.

**Figure 5:**
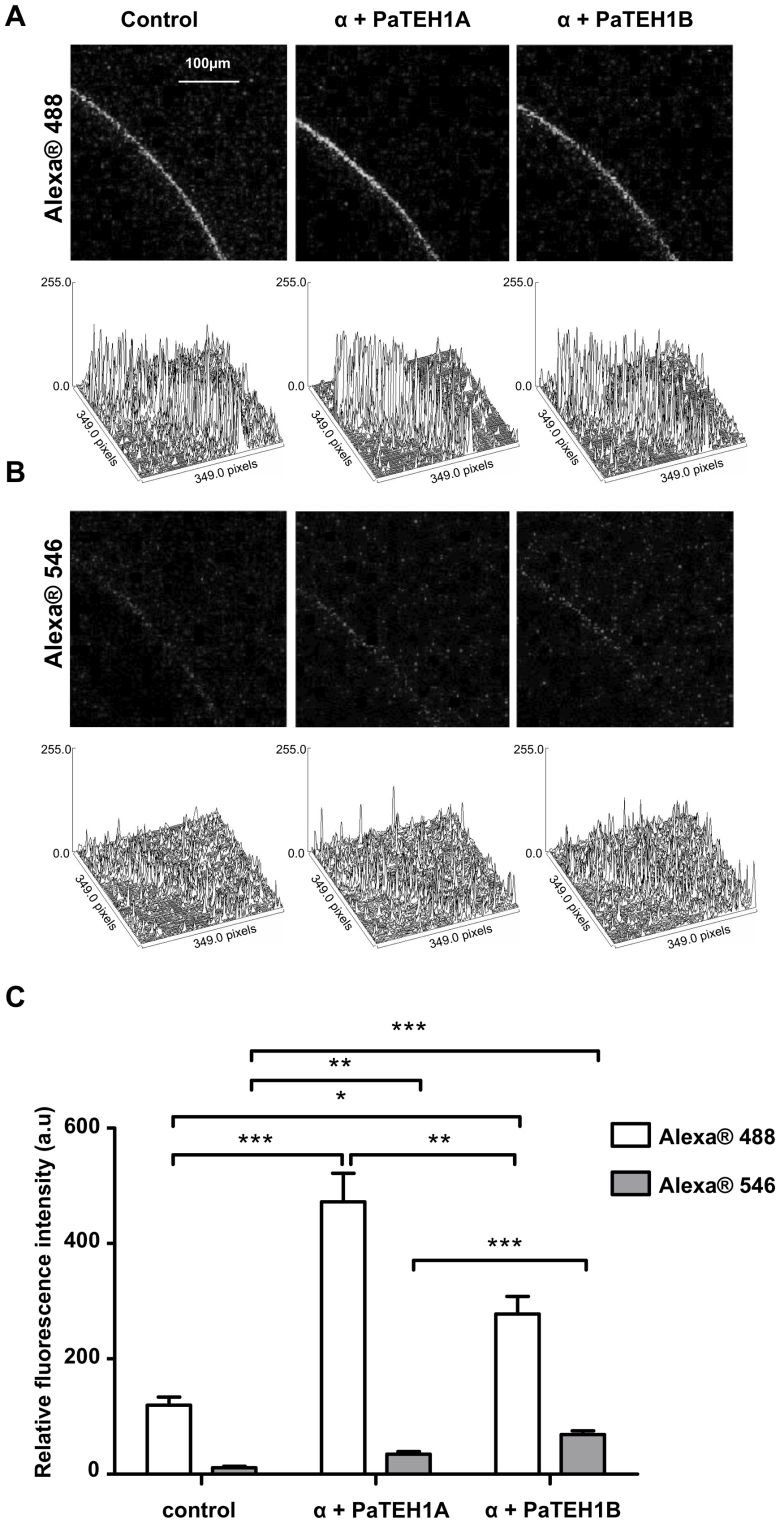
Modulation of Na_v_ channels expression by intron retention mechanism of *teh1*-*like* gene of *P. americana*. Immunofluorescent confocal images of representative oocytes injected with water (control) or with 3 ng of DmNa_v_1.1/PaTEH1A (α+PaTEH1A) or PaTEH1B (α+PaTEH1B) RNAs. A. Immunostaining representative snapshot with an anti Pan-Na_v_ (Alexa®488) antibody and associated wireframe plots drawn with ImajeJ software (version 1.4.3.67). B. Immunostaining representative snapshot with an RGS-His (Alexa®546) antibody and associated wireframe plots drawn with ImajeJ software. C. Relative fluorescence was measured using the ImajeJ software (version 1.4.3.67). For each measurement, an identical region of interest of 5002.2 µm^2^ (292×36 pixels) was defined. Mean relative fluorescence intensity values measured with Alexa®488 (white) and Alexa®546 (grey) are mean ± SEM for 6 oocytes. PaTEH1A shows a stronger expression of the α-subunit than PaTEH1B in oocytes (One-way ANOVA: Alexa®488: F_(2,16)_ = 24.86, p<0.0001 and Alexa®546: F_(2,15)_ = 39.81, p<0.0001, post hoc Tukey test). a.u: arbitrary unit.

In order to assess whether the enhancement of Na^+^ current densities reflect an increase of DmNa_v_1-1 accumulation in *Xenopus* oocytes membrane, we designed two Histag-PaTEH1A and PaTEH1B constructs for double immunostaining. We ensured that the addition of the N-terminal His-tag to PaTEH1A and PaTEH1B did not modify the electrophysiological properties of Na^+^ currents elicited by co-expression with DmNa_v_1-1, even their chaperone-like effects (data not shown). Figure 5A and 5B show immunofluorescent confocal images recorded for oocyte injected with water (negative control) and with DmNa_v_1.1 together with PaTEH1A or PaTEH1B RNAs. The data show a significant increase (1.8 fold, p<0.01, Figure 5C) in the membrane relative fluorescence intensity (Alexa®488 for DmNa_v_1-1) in oocytes expressing DmNa_v_1-1/PaTEH1A compared with oocytes expressing DmNa_v_1-1/PaTEH1B. This indicated that PaTEH1A induced a stronger expression of the DmNa_v_1-1 channels in oocytes membrane than PaTEH1B. Unexpectedly, we also observed a higher level of membrane relative fluorescence intensity (Alexa®546 for PaTEH1A or PaTEH1B) in oocytes injected with DmNa_v_1-1/PaTEH1B than with DmNa_v_1-1/PaTEH1A RNAs (2.2 fold, p<0.001, Figure 5C). These findings demonstrated that the C-terminal extremity encoded by the intronic sequence of PaTEH1B was responsible for the decrease in the density of DmNa_v_1-1 channels, suggesting that the intron retention mechanism is likely involved in the regulation of Na^+^ channels expression in neurons of *P. americana*.

### Biophysical properties of Na^+^ currents are modified by TEH1 variants in *Xenopus* oocytes

**Figure 6:**
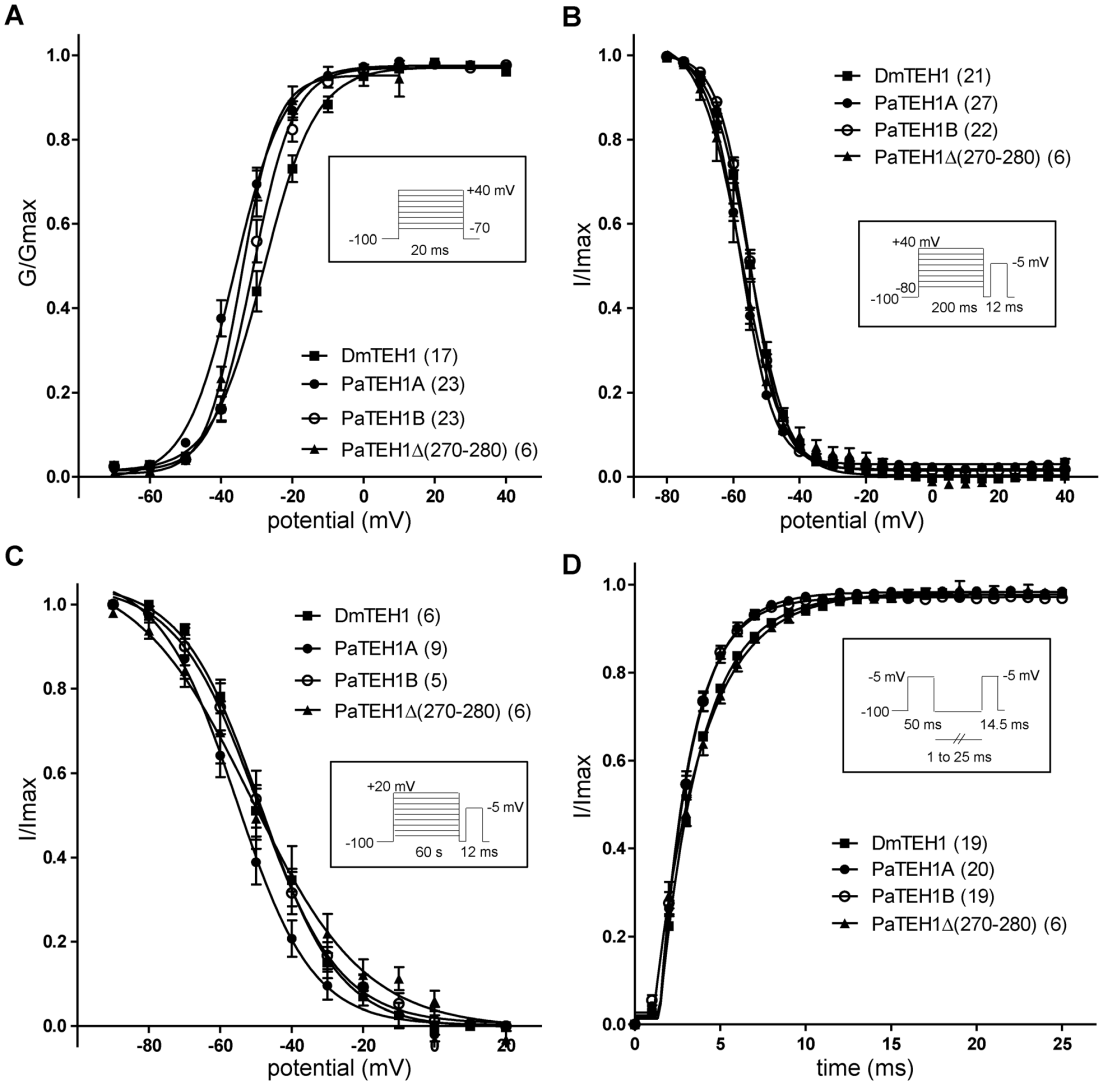
Biophysical properties of Na^+^ currents elicited by co-expression of DmNa_v_1-1 with DmTEH1 or PaTEH1-like subunits. A. Voltage-dependence of activation (One-way-ANOVA: F_(3,65)_ = 6.67, p<0.0005, post-hoc Tukey test). G represents the conductance. B. Voltage dependence of fast steady-state inactivation (One-way-ANOVA: F_(3,75)_ = 6.84, p = 0.0009, post-hoc Tukey test). C. Voltage-dependence of slow steady-state inactivation (One-way-ANOVA: F_(3,23)_ = 4.49, p = 0.014, post-hoc Tukey test). D. Recovery from fast inactivation (One-way-ANOVA: F_(3,62)_ = 2.104, p = 0.1098, post-hoc Tukey test). Na^+^ current amplitudes (I) were measured using the pulse protocols described in the Materials and Methods section and were normalized to the largest current amplitude (Imax). Values are mean ± SEM. The number of individual experiments, each performed with a different oocyte, is indicated in parentheses. Standard protocols are shown in insets.

Activation and inactivation properties of the DmNa_v_1-1 channel co-expressed with DmTEH1, PaTEH1A, PaTEH1B or PaTEH1Δ(270-280) were reported in Table 2. The expression of DmNa_v_1-1 alone led to very small current amplitude either with injection of large amount of RNA (up to 35 ng) and long incubation time (up to 10 days). Thus, we could not accurately assay Na^+^ currents elicited by DmNa_v_1-1 expression alone. Figure 6A shows the voltage-dependence of activation of DmNa_v_1-1 with DmTEH1, PaTEH1A, PaTEH1B or PaTEH1Δ(270-280). We observed a significant negative shift in the voltage-dependence of activation by PaTEH1A compared with DmTEH1 (8.1 mV, p<0.001) and PaTEH1B (5.1 mV, p<0.05) (Table 2). In contrast, the voltage-dependence of activation regulated by PaTEH1B was not significantly different from that of DmTEH1. The voltage-dependences of activation of Na^+^ currents were positively shifted to the same extent by PaTEH1B (3.6 mV, p<0.01) when co-expressed with BgNa_v_1-1a in comparison with PaTEH1A (Figure S3A, Table S1). Interestingly, the voltage-dependence of activation of DmNa_v_1-1 with PaTEH1Δ(270-280) was similar to that of DmNa_v_1-1/PaTEH1A. This indicated that the 11-amino-acid C-terminal end of PaTEH1B modulated activation properties of Na^+^ currents, but not that of PaTEH1A.

**Table 2:** Parameters of voltage-dependence of activation and fast and slow steady-state inactivation for DmNa_v_1.1 co-expressed with DmTEH1, PaTEH1A, PaTEH1B or PaTEH1Δ(270-280) in *Xenopus* oocytes in the presence and absence of lidocaine and DCJW.

	Control		Lidocaine		DCJW	
	V ½ (mV)	k	V ½ (mV)	k	V ½ (mV)	k
*Activation*						
DmTEH1	−28.1±0.6 (17)	7.3±0.5	−22.4±1.6 (5)	8.8±1.5	−26.3±0.9 (8)	7.9±0.8
PaTEH1A	−36.2±0.5 (23)	6.9±0.5	−26.9±2.1 (5)	11.4±1.9	−36.4±1.1 (7)	8.2±0.9
PaTEH1B	−31.1±0.5 (23)	5.8±0.5	−20.6±1.4 (6)	9.2±1.3	−22.4±1.4 (5)	7.3±1.2
PaTEH1Δ(270-280)	−34.6±0.9 (6)	4.9±0.5	−19.9±2.1 (6)	11.9±2.0	−31.7±0.9 (5)	7.3±0.9
*Fast inactivation*						
DmTEH1	−55.1±0.3 (21)	5.7±0.2	−58.1±1.3 (6)	8.9±0.9	−57.4±0.9 (7)	7.5±0.7
PaTEH1A	−57.8±0.1 (27)	4.9±0.1	−61.2±1.4 (6)	9.3±0.8	−61.6±0.7 (8)	6.1±0.5
PaTEH1B	−54.9±0.1 (22)	4.9±0.1	−59.2±1.5 (5)	11.9±0.9	−58.8±0.8 (9)	6.7±0.6
PaTEH1Δ(270-280)	−57.7±0.5 (6)	6.2±0.4	−61.0±1.1 (6)	7.5±0.8	−58.7±1.2 (5)	8.6±0.8
*Slow inactivation*						
DmTEH1	−48.7±1.2 (6)	11.0±1.0	−62.9±1.3 (6)	8.7±1.0	−51.9±1.6 (6)	10.2±1.5
PaTEH1A	−55.5±1.8 (9)	10.7±1.4	−70.5±5.1 (5)	13.2±2.9	−61.2±2.6 (7)	11.6±2.0
PaTEH1B	−49.4±1.8 (5)	11.5±1.6	−59.1±2.6 (5)	15.8±2.1	−60.9±2.0 (5)	11.4±1.7
PaTEH1Δ(270-280)	−50.5±2.3 (6)	15.3±2.3	−66.0±1.6 (6)	9.8±1.3	−54.6±2.8 (5)	12.3±2.1

Values are mean ± SEM derived from the number of individual experiments, each performed with a different oocyte. The number of recorded oocytes is indicated in parenthesis.

Figure 6B and 6C show the voltage-dependences of steady-state fast and slow inactivation. The voltage-dependences of fast inactivation of Na^+^ currents are not significantly different between DmNa_v_1-1/DmTEH1 and DmNa_v_1-1/PaTEH1B, and between DmNa_v_1-1/PaTEH1A and DmNa_v_1-1/PaTEH1Δ(270-280) (Table 2). Compared with PaTEH1B, both PaTEH1A and PaTEH1Δ(270-280) caused a small but significant negative shifts in the voltage-dependences of fast inactivation (2.8 mV and 2.9 mV for PaTEH1A and PaTEH1Δ(270-280), respectively, p<0.01). By contrast, no significant difference in the voltage-dependences of fast inactivation was found between BgNa_v_1-1a/PaTEH1A and BgNa_v_1-1a/PaTEH1B (Figure S3B, Table S1). Also, the voltage-dependence of slow inactivation between Na^+^ currents obtained with DmTEH1, PaTEH1B, or PaTEH1Δ(270-280) was not modified. Interestingly, PaTEH1A shifted the voltage-dependence of slow inactivation to a more negative potential, compared to DmTEH1 (−6.8 mV, p<0.05), PaTEH1B (−6.1 mV, no significant) and PaTEH1Δ(270-280) (−5.0 mV, p<0.05) (Table 2). For all combinations, the rate of recovery as illustrated in Figure 6D, showed no significant difference (One-way ANOVA: F_(3,60)_ = 2.104, p = 0.1098, post hoc Tukey test) between the different TEH1s.

### Blocking effects by Na_v_ channel inhibitors depend on TEH1 subtypes

**Figure 7:**
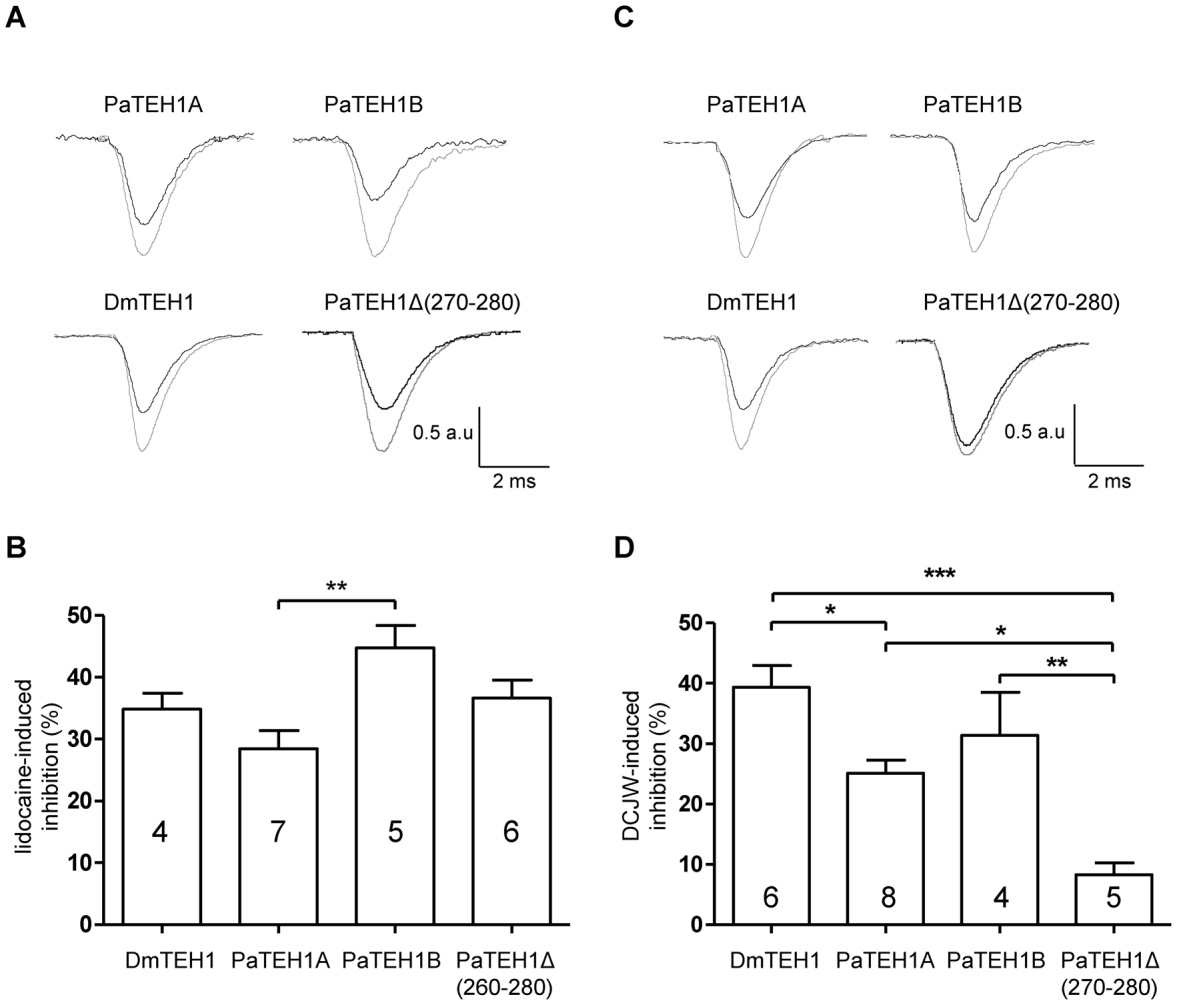
TEH1 isoforms-mediated changes in the sensitivity of DmNa_v_1-1 to Na^+^ channel inhibitors. A. Na^+^ current traces obtained by a 20 ms depolarizing pulse to −10 mV from a holding potential of −100 mV, in the absence (control, grey trace) and in the presence of 2 mM lidocaine at 10 min (black trace). B. Percentage of tonic inhibition of Na^+^ current induced by lidocaine (2 mM, 10 min) for DmNa_v_1-1 co-expressed with DmTEH1, PaTEH1A, PaTEH1B and PaTEH1Δ(270-280) (one-way ANOVA: F_(3,18)_ = 4.85, p<0.0121, post-hoc Tukey test). C. Na^+^ current traces obtained by a 20 ms depolarizing pulse to −10 mV from a holding potential of −100 mV, in the absence (control, grey trace) and in the presence of 2 µM DCJW at 30 min (black trace). D. Percentage of inhibition of Na^+^ current in response to DCJW (2 µM, 30 min) for DmNa_v_1-1 co-expressed with DmTEH1, PaTEH1A, PaTEH1B and PaTEH1Δ(270-280) (one-way ANOVA: F_(3,22)_ = 13.22, p<0.0001, post-hoc Tukey test). Values are mean ± SEM. a.u: arbitrary unit. The number of tested oocytes is indicated in the bar histogram.

**Figure 8:**
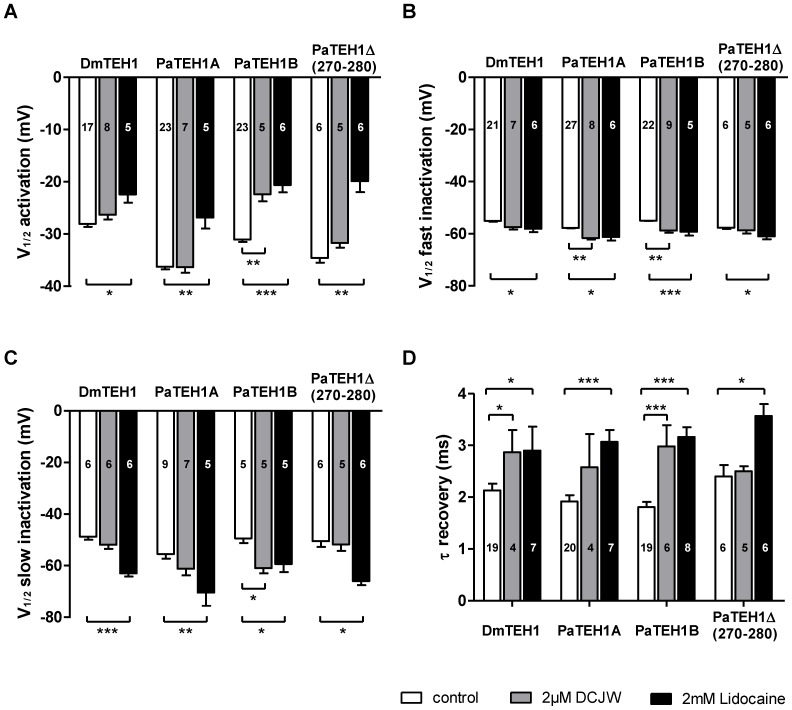
Effects of lidocaine and DCJW on gating properties of Na^+^ currents. Electrophysiological properties of DmNa_v_1-1 co-expressed with DmTEH1, PaTEH1A, PaTEH1B and PaTEH1Δ(270-280) in the absence (white) and the presence of 2 mM lidocaine (black) and 2 µM DCJW (grey). A. Mean data with SEM for V_1/2_ of activation. B. Mean data with SEM for V_1/2_ of fast steady-state inactivation. C. Mean data with SEM for V_1/2_ of slow steady-state inactivation. D. Summary data for recovery time constant from fast inactivation. Statistical tests were performed using a two-tail unpaired t test (control *versus* lidocaine and control *versus* DCJW). The number of tested oocytes is indicated in the bar histogram.

**Figure 9:**
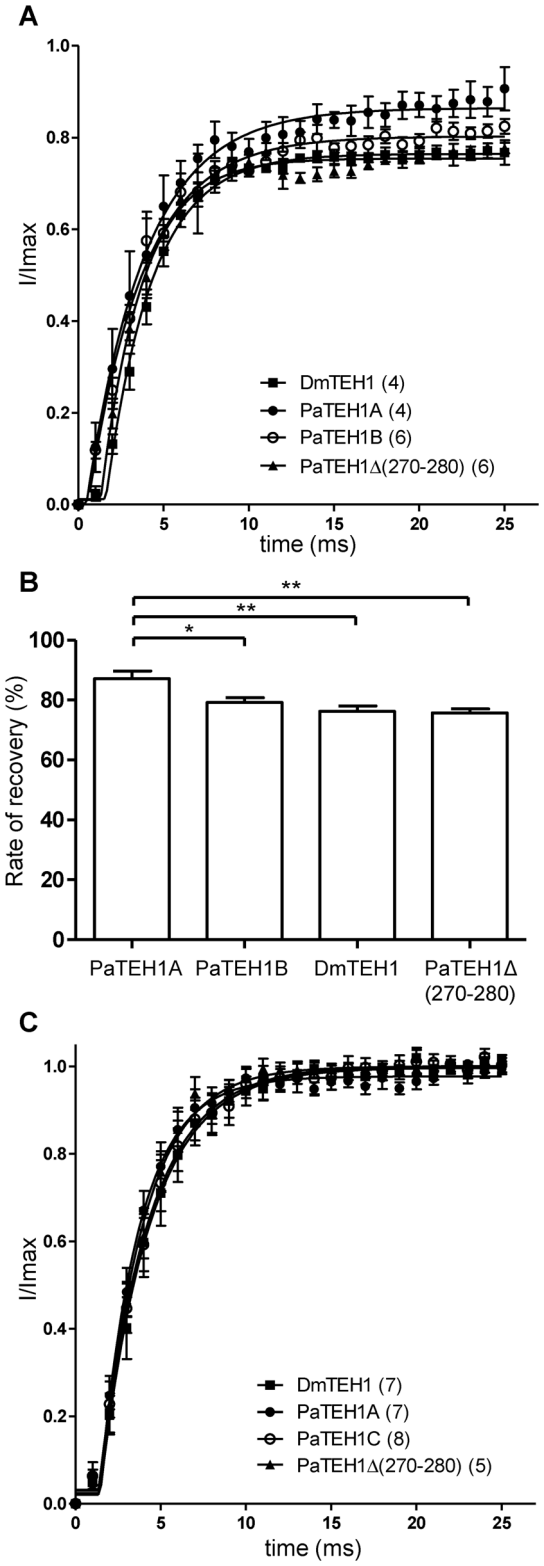
Effects of lidocaine and DCJW on the recovery from fast inactivation of Na^+^ currents. The protocol was the same as shown in Figure 6D. A. Time course of recovery from fast inactivation in the presence of 2 mM lidocaine B. Histogram bars summarizing the effects of 2 mM lidocaine on the rates of recovery from fast inactivation at 20 ms. Data were deduced from the curves shown in A (one-way ANOVA: F_(3,19)_ = 7.71, p<0.0021, post-hoc Tukey test). C. Time course of recovery from fast inactivation in presence of 2 µM DCJW.

We next examined the involvement of the C-terminal end of TEH1 on the pharmacological properties of DmNa_v_1-1 channels. First, we measured the inhibition of Na^+^ currents by the LA, lidocaine (2 mM) (Figure 7A and 7B). Interestingly, lidocaine caused a significant (p<0.01) greater inhibition of Na^+^ currents with PaTEH1B (44.8±3.62%) than with PaTEH1A (28.4±2.97%). No significant difference was observed between DmTEH1 (34.8±2.58%) and PaTEH1A. We also found no significant difference between PaTEH1B and PaTEH1Δ(270-280) (36.6±2.9%). These data indicated that the particular C-terminal end of PaTEH1A (VALLDWEEDRT) played a role in the decrease of the Na^+^ current sensitivity to lidocaine. To understand these differences, we analysed the effect of lidocaine on Na^+^ channel gating properties (Figure 8A–D). Lidocaine significantly shifted the voltage-dependence of activation towards depolarizing potentials in each case (Figure 8A and Table 2) by 9.3 mV (PaTEH1A, p = 0.0036), 10.5 mV (PaTEH1B, p = 0.0005), 5.7 mV (DmTEH1, p = 0.0466) and 14.7 mV (PaTEH1Δ(270-280), p = 0.008) compared to the control (Table 2). Lidocaine also induced a shift of the voltage-dependence of fast inactivation towards hyperpolarizing potentials: −3.4 mV for PaTEH1A (p = 0.0340), −4.3 mV for PaTEH1B (p = 0.0009), −3.0 mV for DmTEH1 (p = 0.0280) and −3.3 mV for PaTEH1Δ(270-280) (p = 0.0396) compared to the control. Similarly, lidocaine induces a shift in the voltage-dependence of slow inactivation towards hyperpolarizing potentials: −15 mV for PaTEH1A (p = 0.0121), −9.7 mV for PaTEH1B (p = 0.0426), −14.2 mV for DmTEH1 (p = 0.0069) and −16.5 mV for PaTEH1Δ(270-280) (p = 0.0101). Lidocaine significantly increased the time constant of recovery from inactivation by 1.2 ms (PaTEH1A, p = 0.0006), 1.4 ms (PaTEH1B, p<0.0001), 0.8 ms (DmTEH1, p = 0.0338) and 1.1 ms (PaTEH1Δ(270-280), p = 0.0115) compared to control. Lidocaine slowed down the recovery of all tested Na^+^ channels combinations to the same extent (One-way ANOVA: F_(3,20)_ = 1.178, p = 0.35, post hoc Tukey test). In addition, in the presence of lidocaine, Na^+^ currents recovered partially, meaning a partial block of Na^+^ channels in the inactivation state (Figure 9A). The recovery of lidocaine-blocked Na^+^ currents was significantly lower (p<0.01) with PaTEH1A (87.08±2.65%), than with DmTEH1 (76.20±1.85%), PaTEH1B (79.21±1.54%) and (PaTEH1Δ(270-280) (75.71±1.29%) (Figure 9B).

We also assessed the sensitivity of DmNav1-1 co-expressed with the four TEH1 subtypes to DCJW, the active metabolite of indoxacarb which is a Na^+^ channel-targeting insecticide [12], [13]. The Na^+^ currents obtained with DmTEH1 were found more sensitive (39.4±3.6% inhibition) to DCJW (2 µM), than those obtained with PaTEH1A (25.1±2.1%, p<0.05) and with the truncated form, PaTEH1Δ(270-280) (8.3±1.9%, p<0.001) (Figure 7C and 7D). By contrast, no significant difference of sensitivities to DCJW was observed between DmTEH1 and PaTEH1B (31.4±7.1%). Thus, our findings indicate that the truncation of 11 amino acid residues at the C-terminal end of TEH1 β-subunits strongly decreased the sensitivity of Na^+^ channels to DCJW.

We also analyzed the effects of DCJW (2 µM) on the gating properties of DmNa_v_1-1 channels. DCJW shifted the voltage-dependence of activation towards depolarized potentials by 8.7 mV (p = 0.0036; Figure 8A, Table 2) only for DmNa_v_1-1 co-expressed with PaTEH1B. It appeared that DCJW slightly shifted the voltage-dependences of both fast and slow inactivation of Na^+^ currents towards more negative potentials (Figure 8B and 8C). A small negative shift by −3.8 mV and −3.9 mV (p = 0.0034 and p = 0.0060) of the voltage-dependence of fast inactivation was observed with PaTEH1A and PaTEH1B, respectively (Table 2). Surprisingly, we observed that DCJW caused a relative large shift of the slow inactivation towards hyperpolarized potentials by −11.5 mV (p = 0.0470), concerning Na^+^ currents obtained with PaTEH1B. DCJW significantly increases the time constant of recovery from fast inactivation by 0.7 ms (DmTEH1, p<0.05) and by 1.1 ms (PaTEH1B, p<0.001) compared to control, but not for PaTEH1A and PaTEH1Δ(270-280) (Figure 8D, Table 3). However, these effects are similar for DmTEH1 and PaTEH1B (p = 0.864, unpaired t test).

**Table 3:** Parameters of recovery from fast inactivation for DmNa_v_1-1 co-expressed with DmTEH1, PaTEH1A, PaTEH1B, or PaTEH1Δ(270-280) in the presence and absence of lidocaine and DCJW.

	Control	Lidocaine	DCJW
	τ (ms)	τ (ms)	τ (ms)
DmTEH1	2.1±0.1 (19)	2.9±0.5 (4)	2.8±0.4 (7)
PaTEH1A	1.9±0.1 (20)	3.1±0.2 (4)	2.6±0.6 (7)
PaTEH1B	1.8±0.1 (19)	3.2±0.2 (6)	2.9±0.4 (8)
PaTEH1Δ(270-280)	2.4±0.2 (6)	3.5±0.2 (6)	2.5±0.1 (6)

Values are mean ± SEM derived from the number of individual experiments, each performed with a different oocyte. The number of recorded oocytes is indicated in parenthesis.

In conclusion, lidocaine caused similar modifications on activation, fast and slow inactivation properties of Na^+^ currents regardless which TEH1 variant is co-expressed. Concerning DCJW, the mode of action of inhibition and its effects on gating properties varied depending on the type of TEH1 variants co-expressed. Like lidocaine, DCJW also slowed the recovery rate from fast inactivation of Na^+^ channels, except for PaTEH1Δ(270-280). Remarkably, lidocaine impaired the total recovery of Na^+^ channels after inactivating pulse, while DCJW did not (Figure 9C). However, DCJW had little effects on the voltage-dependences of activation, fast and slow inactivation. Interestingly, the C-terminal end of PaTEH1 modulated the action of DCJW by affecting the voltage-dependence of activation and the recovery from fast inactivation. Altogether, our findings reveal the involvement of TEH1 variants in modulating the blocking action of lidocaine and DCJW on insect Na_v_ channels.

## Discussion

This study reports for the first time the consequences of an intron retention mechanism of ancillary subunits of insect neuronal Na_v_ channels on regulation of channel expression, gating and sensitivity to sodium channel inhibitors. We found that two TEH1-like variants, PaTEH1A and PaTEH1B, were exclusively and differentially expressed in the nervous system of *P. americana*. While PaTEH1A is a spliced form, PaTEH1B result from an intron retention mechanism converting an undecapeptide to a novel decapeptide at the C-terminal end of the protein. This causes a decrease in the Na^+^ current density and also modifies both gating properties of Na^+^ channels. In addition, our findings pointed out marked differences in the blocking mechanism induced by lidocaine and DCJW. Sensitivities of Na^+^ channels to LAs and PTIs appear to be modulated by TEH1-like variants. Thus, the intron retention process of *teh1-like* genes constitutes a novel mechanism that contributes to the functional and pharmacological diversity of Na_v_ channels in insects.

### Neuronal distribution of TEH1-like subunits

The tissue distribution of PaTEH1 variants matches with that of PaNa_v_1 which has been detected in various tissues with different expression level [6]. However, as reported for DmTEH1 [8], PaTEH1 is exclusively expressed in neuronal tissues, indicating that these two homologous subunits are involved in the regulation of neuronal Na_v_ channels. Because *D. melanogaster* and *P. americana* are phylogenetically distant in high diverse order, we can reasonably predict that TEH1 homologs of other insect species are also exclusively expressed in neuronal tissue and may constitute a subfamily of auxiliary subunits involved in modulation of the neuronal Na_v_ channels.

### Potential conservation of an intron retention event in Na_v_ channel auxiliary subunits of insects and mammals

We showed that *Pateh1* gene displays two exons, exon 1 and exon 2, separated by a short intron sequence, intron 1. Exon 2 is short and encodes the last 11 amino-acid residues of C-terminal end of PaTEH1A variant. The retention of intron 1 results in a short coding sequence with an early stop codon in *PaTEH1B*, leading to a novel C-terminal end. To determine whether a similar intron retention splicing mechanism targeting the C-terminal end of TEH1 occurs in other insect species, we examined available genomic sequences in the GenBank database. *teh1* gene of *D. melanogaster* contains an intron of variable length that disrupts the end of the coding sequence (accession number Dmel_CG12806). Bioinformatic analysis of the genome of *N. vitripennis* (accession number NC_015869) led us to the same conclusion. Two transcript variants have been annotated and they only differ by the end of their 3′ coding sequence (accession number XM_003425719, transcript variant 1 and XM_003425720, transcript variant 2), resulting from the presence of the first 72 bp of the intron 1 of the gene (accession number LOC100123225), and leading to a different 24-amino-acid C-terminal end. Since only one copy of *teh1-like* gene has been found in *N. vitripennis* genome, TEH1-like variants 1 and 2 reported in the GenBank may result from a retention intron mechanism. Blast analysis also revealed the existence of two *teh1* bicistronic genes in the genome of the mosquito, *Culex quinquefasciatus* (accession numbers CpipJ_CPIJ006103 and CpipJ_CPIJ006104). Both genes differ by the length of exon 2 (13 bp and 85 bp). Accordingly, the deduced amino acid sequences of both transcripts (accession numbers XM_001847934 and XM_001847935) differ in their length and C-terminal end. Concerning *D. melanogaster* and *Anopheles gambiae*, only one gene and one transcript have been described (accession numbers Dmel_CG12806 and AgaP_AGAP003797). Nevertheless, the presence of a short coding sequence with an early stop codon in the intron sequences of both genes predicts the possibility of a conserved intron retention process in these insects. Therefore, we suggest that intron retention mechanism might be a common mechanism enabled to generate molecular variability of TEH1-like proteins.

Splicing mechanism by intron retention is relatively rare, occurring at less than 5% in both vertebrates and invertebrates [28], [29]. Two intron retention events have been documented in the transcription of mammalian SCN1B genes. The first concerns the retention of intron 5 of rat SCN1B and does not change the coding sequence [30]. The second mechanism involves the retention of intron 3, as reported in both rat and human SCN1B genes [31], [32]. In this case, like PaTEH1, the intron retention event generates two proteins with distinct C-terminal ends, linked to Na^+^ currents with distinct properties. These findings demonstrate that the functional regulation of Na_v_ channel through alternative splicing of auxiliary subunit genes is a conserved process in mammals and insects.

### Consequences of intron retention mechanism on Na_v_ channel biophysical properties

The unspliced variant PaTEH1B caused a 2.2-fold current density decrease, concomitant with an equivalent α-subunit incorporation increase in the plasma membrane, compared to PaTEH1A. The cytoplasmic C-terminal end of TEH1-like subunits is unlikely involved in the enhanced expression of Na_v_ channels, as evidenced by similar Na^+^ current densities with co-expression of PaTEH1A or the C-terminal truncated construction. On the other hand, the novel C-terminal end of PaTEH1B generated by the intron retention process leads to a decrease in Na^+^ current density. This likely results from a decrease i) in the number of oocyte expressing detectable currents and ii) in α-subunit accumulation in oocyte membranes even though the membrane expression level of PaTEH1B is higher than PaTEH1A. We suggest that the accumulation of PaTEH1B in the membrane is toxic for oocytes. We also cannot rule out that the novel C-terminal end of PaTEH1B interacts with other membrane proteins during its trafficking and thereby decreasing PaTEH1B availability for addressing DmNa_v_1-1 α-subunit to the membrane.

Here, we showed that the activation and inactivation properties of DmNa_v_1-1 depend on the associated auxiliary subunits. In the presence of DmTEH1, PaTEH1A, PaTEH1B or a truncated PaTEH1, lacking the last 11-amino-acid residues, Na^+^ currents exhibit small differences in its voltage-dependences of fast inactivation. This indicates that the C-terminal region of these proteins is not involved in the modulation of fast inactivation. However, while V_1/2_ of slow inactivation is similar with DmTEH1, PaTEH1B and truncated PaTEH1, PaTEH1A shifts the voltage-dependence of slow inactivation in the hyperpolarized direction. Collectively, we assume that the C-terminal extremity of PaTEH1A (VALLDWEEDRT) modulates only slow inactivation properties, while the one of PaTEH1B (YVPLSVHDTR) does not. Concerning the modulation of channel activation properties, with PaTEH1A and truncated PaTEH1, the voltage-dependences of channel activation are not changed, while with PaTEH1B, channel voltage-dependence of activation is shifted to depolarized direction. Thus, the C-terminal extremity of PaTEH1B is implied in the modulation of channel activation. Interestingly, the change of the C-terminal end in rat β1A also leads to a shift of the voltage-dependences of activation and inactivation to more depolarized direction [31]. Collectively, the intron retention mechanism appears to be a novel mechanism that could regulate neuronal excitability by finely modulating expression level, activation and slow inactivation of Na_v_ channels in cockroaches. TEH1 and TEH1-like variants shift the voltage-dependences of both activation and inactivation of Na^+^ currents to more negative potentials when compared with TipE [22]. Moreover, kinetics of activation and inactivation of Na^+^ currents are accelerated with TEH1 (data not shown). Since all studies attempting to characterize Na_v_ channel variants of *D. melanogaster* and *B. germanica* properties were carried out using TipE, we predict that the other subunits may dramatically change the kinetic and gating properties of these variants. The similar consequences of the C-terminal end conversion of TEH1 observed with Na_v_ channels from *D. melanogaster* and *B. germanica* strongly suggest that the modulation of Na^+^ currents by TEH1 splicing does not depend on the α-subunit origin. This argues for a highly conserved function of the auxiliary subunit in insect.

### Consequences of intron retention mechanism on Na_v_ channel pharmacological properties

Tonic block by lidocaine is caused by binding to the resting state of Na_v_ channels with lower affinity [17], [18]. Here, we show that lidocaine binds to *Drosophila* resting Na_v_ channels and induced a marked depolarizing shift of the voltage-dependence of activation, indicating a strong voltage-dependence of lidocaine-block as described previously [18]. This effect can be explained by its interaction with S4 segments of domains III and IV [33]. Positive shift of V_1/2_ of activation by lidocaine has already been reported for mammalian Na_v_1.5 and Na_v_1.8, but not for Na_v_1.7 [34], [35]. Besides, phasic block by lidocaine is explained by preferential binding to inactivated state of Na_v_ channels [18]. According to this statement, our findings show that lidocaine causes a leftward shift of voltage-dependences of both fast and slow inactivation. These are also in good agreement with previous studies showing that the mechanism of lidocaine inhibition operates by stabilizing both fast- or slow-inactivated state [36], [37]. This was reinforced by slower recovery rate of fast inactivation of lidocaine-block DmNa_v_1-1 channels. Unexpectedly, our data show that DmNa_v_1-1 channels do not totally recover under lidocaine block from fast inactivation in steady-state conditions. The impairment of recovery of Na^+^ channels after inactivating pulse has not been yet reported. This indicates that a part of lidocaine-DmNa_v_1-1 remains in inactivated state. More interestingly, our data also report that Na_v_ channels are not equally sensitive to lidocaine depending on TEH1 homologs co-expressed with the same α-subunit. The lower sensitivity of DmNa_v_1-1 towards lidocaine is observed with PaTEH1A, for which a lower part of DmNa_v_1-1 is blocked in inactivated state by lidocaine compared to the other auxiliary subunits. The idea that α/β-subunits interaction crucially determines the pharmacology of insect Na_v_ channels is a coherent assumption as reported for lidocaine effects on mammalian Na_v_ channels [38], [39], [40]. Thus, our findings show that the mechanism of lidocaine channel blockade appears to be similar for both insect and mammalian Na_v_ channels and extend a recently published study [41].

DCJW has no effect on the voltage-dependence of activation of Na^+^ currents elicited by DmNa_v_1-1/DmTEH1 or DmNa_v_1-1/PaTEH1A co-expressions in *Xenopus* oocytes. This is in agreement with previous reports carried out both in transfected *Xenopus* oocytes with German cockroach Na_v_ channel [42] and in native DUM neurons of *P. americana* [13], [14]. Surprisingly, in the presence of PaTEH1B variant, we show that DCJW modifies Na^+^ current activation. Thus, we assume that the modification of activation by DCJW depends on the combination of α-subunit/auxiliary subunits. We also show that DCJW does not affect the voltage-dependence of fast inactivation as already reported [16], [42]. By contrast with previous observations, our data show that DCJW moderately shifts both voltage-dependences of fast and slow inactivation of Na^+^ currents elicited by co-expression of DmNa_v_1-1 with TEH1 homologs. Interestingly, the voltage-dependence of slow inactivation of Na^+^ current elicited by the co-expression of PaTEH1B and DmNa_v_1-1 appears more sensitive to DCJW. This indicates that Na_v_ channels formed by DmNa_v_1-1/PaTEH1B displays distinct slow inactivation property, reinforcing the hypothesis that the C-terminal end generated by intron retention interacts with region of α-subunit involved in slow inactivation.

The idea that LAs and PTIs block Na_v_ channels by similar mechanisms is partially based on evidence that lidocaine and DCJW possibly share a common or overlapping binding site on insect Na_v_ channels [13]. Site-directed mutagenesis experiments have shown that the two key LA-interacting residues identified in mammalian Na_v_ channels are important but not critical for lidocaine binding and mode of action on insect Na_v_ channels [41]. Our findings also point out remarkable differences in the mechanisms by which lidocaine and DCJW block insect Na_v_ channels. While lidocaine induces a marked depolarizing shift of the voltage-dependence of activation, DCJW has a weak effect on activation property. In addition, lidocaine causes more pronounced effects on steady-state fast and slow inactivation than DCJW. Repriming of drug bound DmNa_v_1-1 channels is slow, due to strong stabilizing effects of inactivated state by both molecules, but lidocaine seems to block channel in this state. Thus, lidocaine and DCJW inhibitory effects are not equally sensitive to potential. Finally, lidocaine behaves more like a gating modifier of insect Na_v_ channels with a very complex mechanism.

In conclusion, our work demonstrates that ancillary subunits of insect Na^+^ channels can be subjected to an intron retention process, which constitutes an unanticipated mechanism that modulates the electrical excitability of neurons by tuning expression, gating and pharmacological properties of Na_v_ channels. This modulation mechanism occurs by converting the C-terminal end of TEH1-like proteins in the American cockroach. Our findings strongly suggest that this mechanism could likely happen in other insect species, such as *D. melanogaster*. To confirm this hypothesis, the search of TEH1 variant and the neuronal characterization of *teh1* gene in *D. melanogaster* mutant are required. Additionally, our results clearly indicate that it is no longer tenable to investigate the mechanisms underlying the mode of action of insecticides targeting Na_v_ channels without considering the intricate interactions between the pore-forming and auxiliary subunits. Although this study was focused on PTI, we believe that the examination of auxiliary Na_v_ channel subunits should be extended to other insecticide families such as pyrethroids. Supporting this, recent data have shown that mammalian Na_v_1.3 and Na_v_1.6 channels display different sensitivities to pyrethroids when co-expressed with rat β1 and β2 subunits or alone [43], [44].

## Supporting Information

Figure S1 **Expression of PaNa_v_1 channels with and without auxiliary subunits.** This figure shows the current traces obtained by expressing in *Xenopus* oocytes PaNa_v_1 channels with and without auxiliary subunits (DmTipE, DmTEH1, PaTEH1A and PaTEH1B). In all cases, no voltage-dependent currents could be observed. Family of Na^+^ currents were measured at test potentials of −70 mV to 40 mV from a holding potential of −100 mV. A. No currents were detected after injection of PaNa_v_1 alone (11 ng RNA, 6-days incubation) or with DmTEH1 (13.5–27 ng RNA, 8-days incubation), DmTipE (7.4 ng RNA, 11-days incubation), PaTEH1A (7.4 ng RNA, 10-days incubation) and PaTEH1B (5.5 ng RNA, 3-days incubation).
(PDF)

Figure S2 **Modulation of Na^+^ current densities of BgNav channel by PaTEH1A and PaTEH1B auxiliary subunits.** This figure illustrates the families of Na^+^ currents recorded at various test potentials in *Xenopus* oocytes injected with BgNa_v_1-1 channels alone and with PaTEH1A or PaTEH1B variants. The resulting Na^+^ current densities at −5 mV are plotted in a histogram. A. Expression of BgNa_v_1-1a channels with and without auxiliary subunits. Family of Na^+^ currents were measured at test potentials of −70 mV to 40 mV from a holding potential of −100 mV. B. Na^+^ currents were obtained after injection of 10 ng of mRNAs and 3-day incubation of BgNa_v_1-1a alone or with PaTEH1A and PaTEH1B. Na^+^ current density per ng of injected RNA after 3-days incubation. Results are expressed in µA per nF per ng of injected RNA (One-way ANOVA: F(2,30) = 42.94, p<0.0001 post hoc Tukey test). The number of tested oocytes is indicated in the bar histogram.
(PDF)

Figure S3 **Biophysical properties of Na^+^ currents elicited by co-expression of BgNa_v_1-1a with PaTEH1A or PaTEH1B subunits.** This figure shows the voltage-dependence of activation and fast steady-state inactivation of Na^+^ currents elicited by co-expressing BgNa_v_1-1a with PaTEH1A or PaTEH1B subunits. A. Voltage-dependence of activation. G represents the conductance. B. Voltage dependence of fast steady-state inactivation. Values are mean ± SEM. The number of individual experiments, each performed with a different oocyte, is indicated in parentheses.
(PDF)

Table S1 Boltzmann fits of activation and inactivation curves showed in figure S3 yielded V_1/2_ of activation and inactivation voltages as well as slope factors which are summarized in Table S1.
(PDF)
