# Supplementary material for: Intron Retention in mRNA Encoding Ancillary Subunit of Insect Voltage-Gated Sodium Channel Modulates Channel Expression, Gating Regulation and Drug Sensitivity
Source: PLoS One. 2013 Aug 15;8(8):e67290. doi: 10.1371/journal.pone.0067290 (PMC3744522; doi:10.1371/journal.pone.0067290)
Supplement: Figure S1 — Expression of PaNav1 channels with and without auxiliary subunits. This figure shows the current traces obtained by expressing in Xenopus oocytes PaNav1 channels with and without auxiliary subunits (DmTipE, DmTEH1, PaTEH1A and PaTEH1B). In all cases, no voltage-dependent currents could be observed. Family of Na+ currents were measured at test potentials of −70 mV to 40 mV from a holding potential of −100 mV. A. No currents were detected after injection of PaNav1 alone (11 ng RNA, 6-days incubation) or with DmTEH1 (13.5–27 ng RNA, 8-days incubation), DmTipE (7.4 ng RNA, 11-days incubation), PaTEH1A (7.4 ng RNA, 10-days incubation) and PaTEH1B (5.5 ng RNA, 3-days incubation). (PDF) [file pone.0067290.s001.pdf]

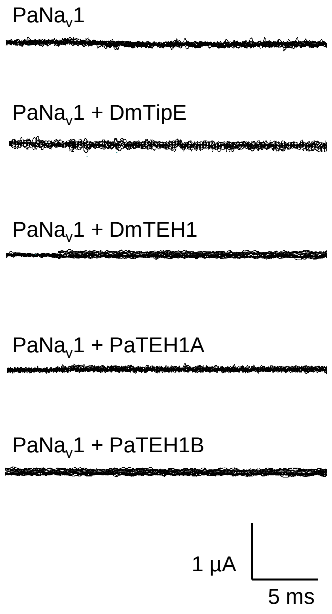
PaNa$_v$1
PaNa$_v$1 + DmTipE
PaNa$_v$1 + DmTEH1
PaNa$_v$1 + PaTEH1A
PaNa$_v$1 + PaTEH1B
1 µA
5 ms
