# Supplementary figures and images for: Intron Retention in mRNA Encoding Ancillary Subunit of Insect Voltage-Gated Sodium Channel Modulates Channel Expression, Gating Regulation and Drug Sensitivity
Source: PLoS One. 2013 Aug 15;8(8):e67290. doi: 10.1371/journal.pone.0067290 (PMC3744522; doi:10.1371/journal.pone.0067290)

**A**

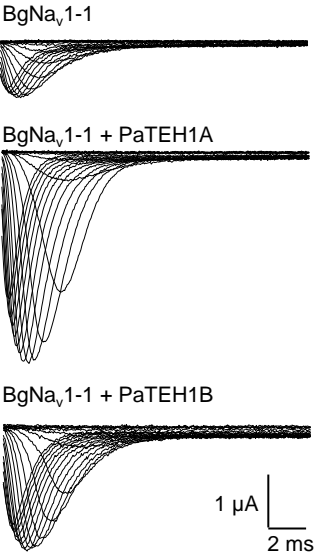

**B**

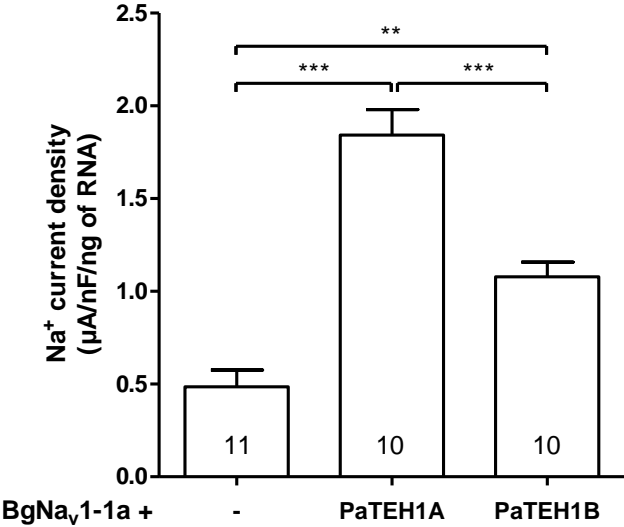

Supplement: Figure S2 — Modulation of Na+ current densities of BgNav channel by PaTEH1A and PaTEH1B auxiliary subunits. This figure illustrates the families of Na+ currents recorded at various test potentials in Xenopus oocytes injected with BgNav1-1 channels alone and with PaTEH1A or PaTEH1B variants. The resulting Na+ current densities at −5 mV are plotted in a histogram. A. Expression of BgNav1-1a channels with and without auxiliary subunits. Family of Na+ currents were measured at test potentials of −70 mV to 40 mV from a holding potential of −100 mV. B. Na+ currents were obtained after injection of 10 ng of mRNAs and 3-day incubation of BgNav1-1a alone or with PaTEH1A and PaTEH1B. Na+ current density per ng of injected RNA after 3-days incubation. Results are expressed in µA per nF per ng of injected RNA (One-way ANOVA: F(2,30) = 42.94, p<0.0001 post hoc Tukey test). The number of tested oocytes is indicated in the bar histogram. (PDF) [file pone.0067290.s002.pdf]

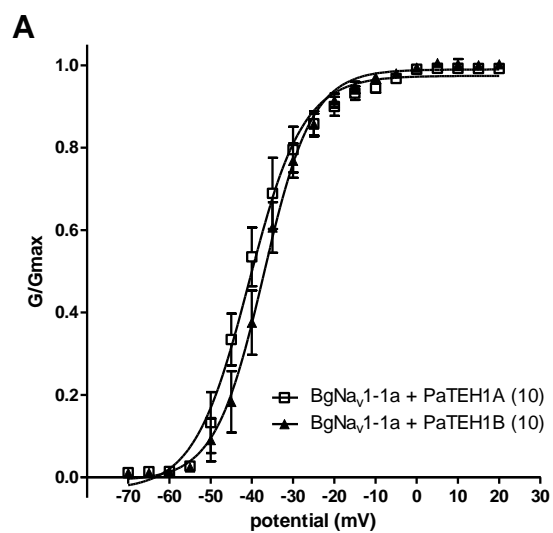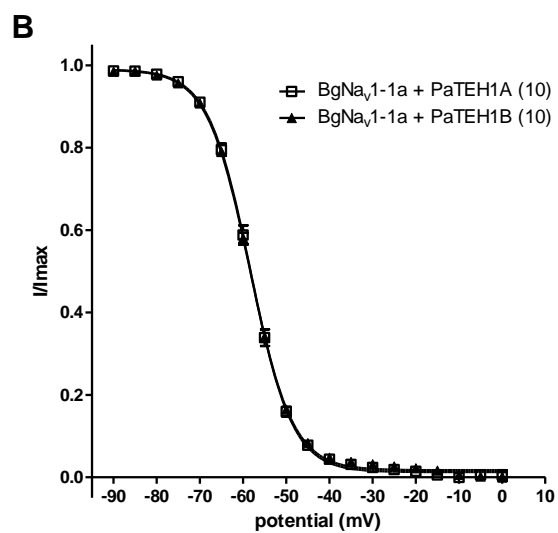

Supplement: Figure S3 — Biophysical properties of Na+ currents elicited by co-expression of BgNav1-1a with PaTEH1A or PaTEH1B subunits. This figure shows the voltage-dependence of activation and fast steady-state inactivation of Na+ currents elicited by co-expressing BgNav1-1a with PaTEH1A or PaTEH1B subunits. A. Voltage-dependence of activation. G represents the conductance. B. Voltage dependence of fast steady-state inactivation. Values are mean ± SEM. The number of individual experiments, each performed with a different oocyte, is indicated in parentheses. (PDF) [file pone.0067290.s003.pdf]
