# Supplementary material for: Intron Retention in mRNA Encoding Ancillary Subunit of Insect Voltage-Gated Sodium Channel Modulates Channel Expression, Gating Regulation and Drug Sensitivity
Source: PLoS One. 2013 Aug 15;8(8):e67290. doi: 10.1371/journal.pone.0067290 (PMC3744522; doi:10.1371/journal.pone.0067290)
Supplement: Table S1 — Boltzmann fits of activation and inactivation curves showed in figure S3 yielded V1/2 of activation and inactivation voltages as well as slope factors which are summarized in Table S1. (PDF) [file pone.0067290.s004.pdf]

Table S1. Voltage-dependence of activation and fast inactivation of $BgNa_v1$-1a with or without PaTEH1A or PaTEH1B.

Na_v channel	Activation		Fast inactivation		n
	$V_{1/2}$ (mV)	k (mV)	$V_{1/2}$ (mV)	k (mV)	
$BgNa_v1$-1a	-25.7 ± 0.3	6.6 ± 0.3	-48.8 ± 0.2	5.0 ± 0.2	15
BgNav1-1a + PaTEH1A	-40.8 ± 0.8	6.6 ± 0.7	-58.3 ± 0.2	4.9 ± 0.1	10
BgNav1-1a + PaTEH1B	-37.2 ± 0.6	5.9 ± 0.5	-58.4 ± 0.1	4.9 ± 0.1	10

The voltage-dependence of activation and fast inactivation data were fitted with Boltzmann equations to determine $V_{1/2}$, the voltage for half-maximal activation and k, the slop factor for activation or fast inactivation. Data represents mean ± SEM.
